# Weak latitudinal trends in reproductive traits of Afromontane forest trees

**DOI:** 10.1093/aob/mcad080

**Published:** 2023-07-05

**Authors:** R C Swart, S Geerts, C J Geldenhuys, J Pauw, A Coetzee

**Affiliations:** Department of Conservation Management, Faculty of Science, George Campus, Nelson Mandela University, George 6530, South Africa; Department of Conservation and Marine Sciences, Cape Peninsula University of Technology, PO Box 652, Cape Town, 8000, South Africa; Department of Plant and Soil Sciences, University of Pretoria, Pretoria 0184, South Africa; Department of Conservation Management, Faculty of Science, George Campus, Nelson Mandela University, George 6530, South Africa; Department of Conservation Management, Faculty of Science, George Campus, Nelson Mandela University, George 6530, South Africa

**Keywords:** Afromontane forest, latitude, pollination syndromes, dispersal syndromes

## Abstract

**Background and Aims:**

Is the increase in species diversity patterns towards lower latitudes linked to reproductive traits? Plant reproductive organs influence reproductive isolation and hence species divergence. Abiotic differences between temperate and tropical regions can also directly impact on plant reproductive traits. Here we provide a novel overview of southern hemisphere, Afromontane forest tree taxonomical patterns and ask whether reproductive traits relate to latitude, while accounting for environmental (tree height) and evolutionary (biogeographical affinity) selective forces.

**Methods:**

We compiled a novel dataset with (1) flower colour, size and pollination syndrome and (2) fruit colour, size and dispersal syndrome for 331 tree species found in six Afromontane forest regions. We categorized each species into latitudinal distribution using these six regions, spanning the southern Cape (34º S) to Mount Kenya (0º S). Additionally, we gathered maximum tree height (m) for each species and determined the global distribution of all 196 tree genera (Afrotropical, Palaeotropical or Pantropical).

**Key Results:**

Species, genera and families showed a general decrease in richness away from tropical and subtropical forests towards warm temperate forests. Southern Afrotemperate forests (the furthest south) had the highest tree endemism. There was no relationship between latitude and the reproductive traits tested here. Biogeographical affinity related to fruit colour and dispersal syndrome, with palaeotropical genera showing relative increases in black-purple fruit colour compared with pantropical genera, and palaeotropical genera showing relative increases in biotic seed dispersal compared with Afrotropical genera, which showed higher relative abiotic seed dispersal. Taller trees had a higher chance to be wind or insect pollinated (compared with bird pollinated) and had larger fruits.

**Conclusions:**

Latitude explained patterns in Afromontane tree taxonomic diversity; however, tree reproductive traits did not relate to latitude. We suggest that phylogenetic conservatism or convergence, or both, explain the reported patterns.

## INTRODUCTION

Latitude and its effects on biodiversity are among the more widely debated ecological topics (Gaston *et al*., 1998; Gaston, 2000; Willig *et al*., 2003) but it is still poorly understood how species interactions are affected by changes in latitude (Moles and Ollerton, 2016). Flowers and fruits are the main reproductive organs of trees and are under strong selection pressure, both biotic and abiotic, to enhance pollination and seed dispersal, respectively (Delmas *et al*., 2020; Valenta and Nevo, 2020). Floral and fruit traits vary between tree species and can be both a reflection of past evolutionary selection and more recent environmental pressures (Sobral *et al*., 2015; Sauquet *et al*., 2017; Delmas *et al*., 2020). For example, phylogenetic conservatism can lead to trait homogeneity within certain phylogenies (e.g. Chilean rain forest Myrtaceae) (Smith-Ramírez *et al*., 1998; Vasconcelos *et al*., 2018). Alternatively, trait homogeneity across phylogenies could indicate selective pressures towards adaptive convergence. For instance, a dominance of whitish flowers in Australian rainforests is found across trees with different biogeographical affinities and could reflect regional adaptation (Delmas *et al*., 2020).

Plant reproductive organs are often involved in reproductive isolation and hence species divergence. Predictably, there is a link between species diversity and reproductive trait diversity. Latitudinal patterns in pollinator and frugivorous animal diversity, which are known to increase towards the tropics (Hawkins *et al*., 2007; Davies and Buckley, 2011; García-Robledo *et al*., 2020), could indirectly reflect associated plant reproductive trait diversity. Flower colour of angiosperms is partially selected upon for its detectability through the visual systems of the pollinator community, largely insects [>80 % of flowering plants are entomophilous (Ollerton *et al*., 2011)], but also larger vertebrates, such as birds and mammals (Shrestha *et al*., 2013; Song and Lee, 2018). Fruit colour, in the case of fleshy fruits that are consumed by animals as primary seed dispersers, is similarly selected upon for its detectability, although through the visual systems of the frugivore community, largely birds and mammals (Nevo *et al*., 2018; Sinnott-Armstrong *et al.*, 2021). There is an evolutionary link between fruit colour diversity and the number of fruit-eating birds present in an ecosystem (Willson and Thompson, 1982), as well as between flower colour and pollinator diversity, the latter expected to decrease away from the tropics (Dalrymple *et al*., 2015). It has been suggested that bird dispersal can select for smaller fruit, compared with mammal dispersal (Valido *et al*., 2011). The implications are that tropical latitudes, which host a richness of frugivorous primates and bats exceeding that of temperate latitudes (Fleming *et al*., 1987), might see a relative increase in fruit size. Similarly, flower and fruit colour could be expected to increase in morphological diversity at tropical latitudes.

Animals are not always the primary selective drivers of flower and fruit colour (Sinnott-Armstrong *et al*., 2021). For example, anthocyanins in plants produce the black, blue, purple and red colours, and their production is affected by exposure to UV radiation (Chalker-Scott, 1999; Steyn *et al*., 2002; Gould, 2004), which increases nearer to the equator (Beckmann *et al*., 2014). Strong relationships are shown between plant reproductive traits and climatic gradients, with fleshy fruits increasing in proportion to non-fleshy fruits at lower latitudes in Australia (Chen *et al*., 2017). Thus, apart from frugivore selective pressures, physiological responses to the external environment can also impact on fruit traits (Valenta *et al*., 2018).

Fruit colour has been shown to be related to latitude, as gathered from numerous studies (Willson *et al*., 1990), with temperate latitudes dominated by red and black fruits (predominantly bird dispersed) and colours yellow, green and brown increasing in relative abundance at lower latitudes. Also, flowers could have higher colour diversity at higher latitudes, linked to fewer pollinators and lower precipitation further from the equator (Dalrymple *et al*., 2015). Alternatively, floral disparity, or the morphological diversity of flowers, has been shown to be greatest at tropical latitudes for the order Ericales, which was, importantly, not coupled with plant species richness patterns (Chartier *et al*., 2021). Of further interest at latitudinal scales are the proportional changes in dispersal and pollination syndromes, the former shown to differ at the continental scale (Dugger *et al*., 2019; Delmas *et al*., 2020) and the latter shown to differ at continental (Aizen and Ezcurra, 1998) and regional (Chazdon *et al*., 2003) scales. For example, Afrotemperate forests in KwaZulu–Natal, South Africa, had a higher incidence of wind pollination compared with scarp and coastal forests, which were linked to steep topography, more seasonal droughts and less pollinator diversity in Afrotemperate forests (Griffiths and Lawes, 2006). Notably, at temperate latitudes, abiotic dispersal can be expected to increase relative to tropical latitudes (Chen *et al*., 2017).

In the tropics, where high species densities are expected to be packed into narrow ecological niches (Mittelbach *et al*., 2007; Ollerton, 2012), specialization might be expected to increase, which could impact on pollination and dispersal syndromes. Unexpectedly, it has been shown that pollinator specialization does not correlate with latitude (Ollerton and Cranmer, 2002), with later work indicating exceptional specialization in temperate regions, i.e. the Cape Floristic Region *sensu lato* (Geerts and Pauw, 2009; Geerts, 2011; Schleuning *et al*., 2012; Pauw and Stanway, 2015). However, there are almost no data to support or refute it and therefore the idea that specialization and related reproductive traits are influenced by latitude remains contentious at best (Vizentin-Bugoni *et al*., 2018).

The Afromontane flora lends itself to testing how latitude influences plant reproductive traits within one vegetation zone, because it spans both temperate and tropical latitudes. Africa is the only continent that extends north and south of the bisecting equator to nearly equal distances (roughly up to the 35º latitudinal degree). The flora of the Afromontane archipelago (*sensu*White, 1978), specifically, reflects a highly endemic phytochorion supporting >4000 species, of which >75 % are endemics (White, 1981). These form small pockets of fragmented forests across the continent. Afromontane forests, despite their fragmented nature and the high isolation between forests, share tree species, genera and families, often covering relatively high-altitude mountains or mountain ranges surrounded by lower-altitude – and different – floras. The Afromontane flora is distinct from the Congo rainforest flora, forming scattered forest communities both south (White *et al*., 2001) and north (Chapman and Chapman, 2001) of the Congo basin. It is also floristically distinct from Miombo woodlands (White, 1978).

Afromontane forest trees are seemingly highly dependent on animals for both pollination and seed dispersal, based on the dominance of trees producing small, white flowers (insect pollination) and fleshy fruits (mammal and bird dispersal) (Geldenhuys, 1993; White *et al*., 2001; Venter, 2011; Hyde *et al*., 2021). Given the disjunct, and often isolated, nature of Afromontane forest flora, understanding to what extent animals aid recruitment and gene flow in this flora is of great conservation significance. Many Afromontane forests harbour narrow endemics (De Klerk *et al*., 2002; Fjeldså and Bowie, 2008), some of which are critically endangered (Obunga *et al*., 2022). Africa’s complex topography and vegetation palaeohistory, especially relating to its southern and eastern mountainous regions (Assèdé *et al.*, 2021), greatly shaped the diversity distributions of both pollinators and frugivores in Afromontane forest systems. Latitude is expected to strongly exert selection pressures on tree reproductive traits, indirectly (pollinator and frugivore diversity) and directly (plant physiology).

Here, we determine whether Afromontane forest tree species’ reproductive traits show relation to latitude, accounting for environmental (tree height) and evolutionary (biogeographical affinity) selective forces. We compiled a novel, comprehensive dataset of tree species occurring in six Afromontane forests, spanning the southern Cape (34ºS) to Mount Kenya (0ºS), which includes, per species, (1) flower colour, size and pollination syndrome, and (2) fruit colour, size and dispersal syndrome.

We hypothesize that

(1) tree species richness will decrease away from tropical latitudes,
(2) flower and fruit colour will decrease in morphological disparity away from tropical latitudes,
(3) flower and fruit size will decrease away from tropical latitudes, and
(4) wind pollination and abiotic dispersal will increase away from tropical latitudes.

## MATERIALS AND METHODS

### Study area and dataset

**Fig. 1.**
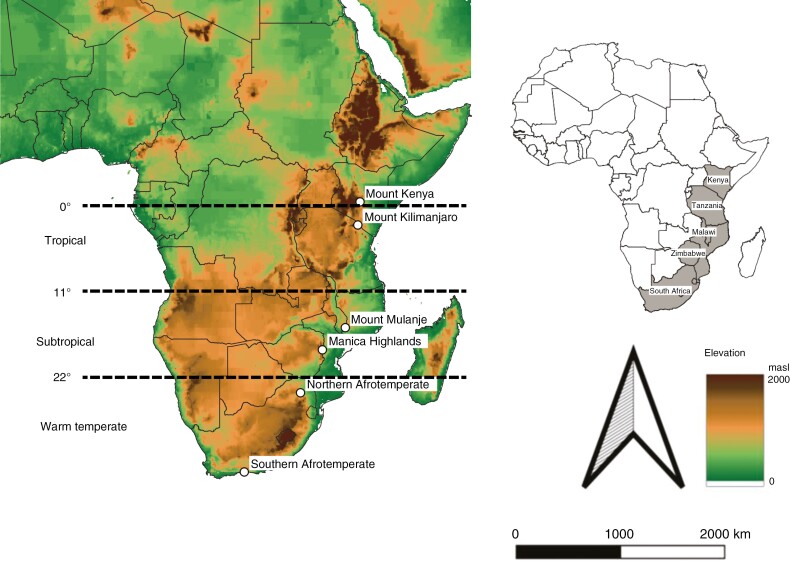
The six Afromontane forest regions included in this study, spanning from the southern Afrotemperate (34ºS) forests to the forests at Mount Kenya (0º).

The dataset includes tree species occurrence from six Afromontane forest regions from the eastern and southern rift mountains, Manica highlands and the Drakensberg range (Fig. 1). The six forests include the southern Afrotemperate forests, northern Afrotemperate forests, Manica Highlands, Mount Mulanje, Mount Kilimanjaro and Mount Kenya. We selected the six forests based on their distribution along the latitudinal gradient and the availability of comprehensive species lists. The six forests are found on a diversity of substrates of varying geological origins (Supplementary Data Table S1). In geological terms, Mount Kilimanjaro and Mount Kenya are recent in origin (<6 mya) compared with the relatively ancient mountain ranges of the Manica Highlands and those underlying southern and northern Afrotemperate forests (>300 mya) (Supplementary Data Table S1). The Afromontane archipelago, as noted by Grimshaw (2001), is not a geographical concept; rather, it is defined by its floristics. We therefore did not consider substrate or age in selecting study forests.

The Afromontane flora contains many endemics, but also many ecological transgressors that occur in two or more major phytochoria or in at least two vegetation types (White, 1978). Also, as pointed out by Linder *et al*. (2005), the distinction between Afromontane flora and surrounding lowland forest flora, especially in the tropics, is often not clear, with a gradual transition rather than a sharp boundary. Viewing the Afromontane flora holistically, we include species that are not only considered as ‘core Afromontane species’, but also species that are associated with Afromontane forests (i.e. *Afrocanthium mundianum*, *Celtis africana*, *Ekebergia capensis*) or those that occur along Afromontane forest margins (i.e. *Buddleja saligna*, *Lachnostylis hirta*, *Virgilia* spp.) in the six study forests.

Only species with woody growth forms, described as a free-standing tree (climbers and spreading shrubs and subshrubs excluded), excluding monocots, and which are found in Afromontane forests or forest margins in the six indigenous forest regions were considered. Notable Afromontane species that were excluded are *Dracaena* spp. (woody monocots) and *Yushania alpina* (non-woody monocot). Plant species <4 m in height when mature were not classified as trees and excluded.

Data were obtained from Geldenhuys (1993), Venter (2011) and Von Breitenbach (1974, 1985) for the list of southern Afrotemperate species. Here, the southern Afrotemperate forest species were confined to the area between the Gouritz River in the west, the Kromme River in the east, the Cape Fold Mountains to the north and the Indian Ocean to the south (Geldenhuys, 1993). The northern Afrotemperate forests are less well defined geographically, and here include the Marakele Afromontane forests (Van Staden *et al*., 2021) and the Northern Mistbelt forests (Limpopo and Mpumalanga Mistbelt forests), which comprise numerous forest subtypes of Afrotemperate affinity (Geldenhuys, 1992; Mucina *et al*., 2006; Lötter *et al*., 2014). We consulted species lists obtained from Mucina *et al*. (2006), Van Staden *et al*. (2021) and Geldenhuys (1992) for the list of northern Afrotemperate species. The Manica Highlands here include the Bvumba, Chimanimani and Nyanga indigenous forest complexes, with species records obtained from the online, open access resource *Flora of Zimbabwe* (Hyde *et al*., 2021) and a species list of moist forests compiled by Timberlake *et al*., (2021). The forest tree and large shrub species around the Mulanje massif were documented by White *et al*. (2001) and this publication was used to compile the list of species from Mount Mulanje. For tree species from Mount Kilimanjaro, we used species lists from Lovett *et al*. (2000) and Hemp (2006), with Zhou *et al*. (2018) used for the tree species found at Mount Kenya. Additionally, we used the online platform GBIF to supplement the data (GBIF.org, 2021), manually filtering species records by excluding non-native and/ or planted trees. Thus, only naturally occurring, indigenous species were included. We are confident the number of tree species recorded accurately reflects the number of species and is not an effect of missing data since larger number of species were recorded for regions with less data availability.

### Latitudinal distribution, tree height and biogeographical affinity

To determine the relation between reproductive traits and latitude, we divided the six forest regions into three latitudinal distribution categories (Delmas *et al*., 2020), namely tropical (Mount Kenya, Mount Kilimanjaro), subtropical (Mount Mulanje, Manica Highlands) and warm temperate (northern Afrotemperate, southern Afrotemperate). Subsequently, we categorized each tree species into one of the following six latitudinal categories: tropical (Mount Kenya and/or Mount Kilimanjaro only); subtropical (Mount Mulanje and/or Manica Highlands only); warm temperate (northern Afrotemperate and/or southern Afrotemperate only); tropical to warm temperate (species across whole range); tropical to subtropical (species limited to tropical and subtropical forests); and subtropical to warm temperate (species limited to subtropical and warm temperate forests).

For each species in the dataset, we determined its biogeographical affinity based on the present geographical distribution of its genus (Supplementary Data Tables S2 and S3). Regions of biogeographical affinity to which genera were ascribed to include Austral (genus distributed on multiple southern hemispheric continents), Afrotropical realm (distributed in Africa south of the Sahara, the Arabian Peninsula and Madagascar, including endemic Afromontane genera), cosmopolitan (distributed on most continents), Mediterranean (predominantly distributed in Mediterranean regions), palaeotropical (confined to tropical regions of Africa and/or Asia including the Pacific) and pantropical (confined to tropical regions of Africa, America and/or Asia including the Pacific). Maximum tree height (metres) was used in analyses (Supplementary Data Tables S2 and S3).

### Flower colour, size and pollination syndrome

We categorized each species’ flower colour based on the dominant ‘human vision’ colour of the flower. Colour categories included white (including white, pale white to cream, and greenish-yellowish white), green, yellow, orange-red and pink-purple. Conifers and trees in the *Ficus* genus were placed in a separate category, termed ‘no external flowers’, due to the absence of flowers in the former and the lack of external flowers in the latter. Flowers displaying more than one colour, having young flowers change colour with age, or in which females and males show sexual dimorphism were placed in the category of the dominant colour. For flower size, we used the largest measurement of the apparent display organ (in millimetres), which included mostly petals but also other organs in some species, such as sepals. This was obtained from exhaustive literature scans (Supplementary Data Tables S2 and S3).

We categorized each tree species into the following pollination syndromes: ‘bird’, ‘insect’, ‘bird–insect’ and ‘wind’ pollinated. Bird-pollinated species were grouped as such when flowers are either large, striking, bright orange-red, tubular or a combination of these characteristics, including confirmation from the literature (Supplementary Data Tables S2 and S3). Flowers that are small, inconspicuous, creamy-white to green, scented or a combination of these characteristics were grouped as insect pollinated. To support this, for each tree or shrub species a literature scan was done, specifically looking for pollination syndrome or confirmed pollinator (Supplementary Data Tables S2 and S3). The category ‘bird–insect’ included species possibly pollinated by both insects and birds, with no one pollination method being clear from its flower morphology or the literature. For four species, we were unable to obtain information on flower morphology or pollination syndromes. In these cases, we ascribed the mode of pollination using other, closely related species within the genus, for which pollination is known. This includes the species *Maytenus albata* (insect pollinated), *Scolopia oreophila* (insect pollinated) and *Tricalysia acocantheroides* (insect pollinated).

### Fruit colour, size and dispersal syndrome

We categorized each species’ fruit into colour (fleshy fruit only), which included the dominant human vision colour of the fruit. Colour categories were black-purple, brown, green, orange, red and yellow. Species with fruits displaying more than one colour, having young fruits that change colour as they mature, having fruit appendages of differing colours, or having fruits showing a range of colour were placed in the category of the dominant colour, or the colour when fruits are ripe, as determined by exhaustive literature scans (Supplementary Data Tables S2 and S3). For each tree species, the length and width of fruit were obtained (in millimetres), with the longest measurement used in data analyses (for instance, if a fruit size was 6 × 8 mm, 8 would be used).

We further ascribed each tree species to seed dispersal syndromes: ‘biotic’, ‘abiotic’, or ‘multiple’ dispersed. ‘Biotic dispersal’ comprised species for which a fleshy fruit, or an aril, covers the whole or parts of the seed. This also included those taxa for which we could find literature confirming it as animal dispersed (Supplementary Data Tables S2 and S3). We categorized taxa producing grain-like, small seeds in woolly hairs (e.g. *Brachylaena* spp.), seeds that have explosive, dehiscent mechanisms with wing-like structures (e.g. *Catha edulis*) or those that had reference to being wind or gravity dispersed in the literature in the ‘abiotic dispersal’ category (Supplementary Data Tables S2 and S3). Only one species was determined to be a probable water disperser, *Hibiscus burtt-davyi*, and this was placed in the ‘abiotic dispersal’ category. ‘Multiple dispersal’ was ascribed to species as follows: (1) seeds that are produced in pods, notably in the family Fabaceae [excluding Fabaceae members where adaptations for either a biotic or abiotic dispersal method is clear, as in *Craibia brevicaudata*, which has explosive pods for gravity dispersal (‘abiotic dispersal’)], or (2) those with no one clear method of dispersal but more than one possibility, e.g. dehiscent fruits followed by secondary dispersal by animals. This category also included taxa with known multiple dispersal methods, e.g. *Combretum kraussii*, which has seeds dispersed by wind and/or animals, and *Clutia pulchella*, which has seeds with lipid-rich elaiosomes, attracting ants as secondary dispersers (Supplementary Data Tables S2 and S3). For three species, we were unable to confidently determine its dispersal syndrome: *Lepidotrichilia volkensii*, *Necepsia castaneifolia chirindica* and *Mallotus oppositifolius.* These were removed from dispersal syndrome analyses.

### Statistical analyses

To determine the similarity in tree species composition, cluster analysis between the six forest regions was done in PRIMER 6, indicating similarity of tree species assemblages (Clarke and Gorley, 2006). Analyses were done on square-root-transformed data, with Bray–Curtis similarity measures applied prior to analysis. To determine whether latitude correlates with tree reproductive traits, we fitted both generalized linear mixed-effect models (GLMMs, for categorical response variables) and linear mixed-effect models (LMMs, for numerical response variables) using the *lme4* package (Bates *et al*., 2014) in R (R Core Team, 2019). Four respective GLMMs contained the response variables ‘flower colour’, ‘pollination syndrome’, ‘fruit colour’ and ‘dispersal syndrome’, with each model containing the variables latitudinal distribution (categorical), tree height (numerical) and biogeographical affinity (categorical). The family of each species was included as a random variable. GLMMs were fitted using the binomial family distribution. Two respective LMMs contained the response variables ‘flower size’ and ‘fruit size’ (both followed a positive skew non-normal distribution and were log-transformed), with each model containing the variables latitudinal distribution (categorical), tree height (numerical) and biogeographical affinity (categorical). The family of each species was included as a random variable.

Due to the negligible contributions and the disjunct nature of the biogeographical affinities Mediterranean (*n* = 5), Austral (*n* = 2) and Cosmopolitan (*n* = 12), species with these three affinities were removed from statistical analyses in which the factor ‘biogeographical affinity’ was used. Tree species with no external flowers were excluded from flower colour and flower size analyses (mostly the conifers and *Ficus* spp.). Pollination syndrome analyses again included these tree species. For pollination syndrome analysis, the categories ‘bird’ and ‘bird–insect’ were combined into the category ‘bird–insect’ due to low numbers recorded of exclusively bird-pollinated tree species (*n* = 4). Tree species with non-fleshy fruit were excluded from fruit colour and fruit size analyses. Dispersal syndrome analyses again included these species.

## RESULTS

### Taxonomical overview and trends

A total of 331 tree species, from 73 families and 196 genera, were documented. The most speciose families were Rubiaceae (51), Celastraceae (17), Fabaceae (14), Salicaceae (13) and Euphorbiaceae and Rutaceae (both 12). The most speciose genera were *Psychotria* (8), *Ficus* (7), *Searsia* (7), and *Allophylus*, *Canthium*, *Dovyalis*, *Maytenus* and *Pavetta* (all 5). A total of 27 tree species occurred in all six forest regions. Of these, 24 are insect pollinated, one is bird pollinated (*Halleria lucida*) and two are wind pollinated (*Afrocarpus falcatus*, *Podocarpus latifolius*). Of these 27 species, only *Trichocladus ellipticus* is dispersed through abiotic means, the rest biotically.

**Fig. 2.**
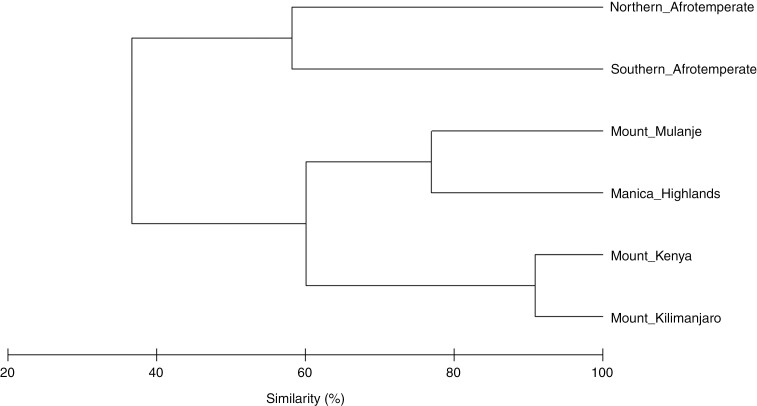
Cluster analysis revealing percentage similarities in the species composition of six Afromontane forests between the latitudinal degrees 34º (southern Afrotemperate) and 0º (Mount Kenya).

The number of species, genera and families decreased away from the tropics (Table 1). The number of families decreased from between 60 and 62 from the Manica Highlands northwards to 43 in southern Afrotemperate forests. Southern Afrotemperate forests had the highest proportion of endemic species (27.78 %), followed by northern Afrotemperate forests (12.40 %) and Mount Mulanje (8.97 %) (Table 1). Cluster analysis revealed that forests group together based on latitude, with a clear tropical (Mount Kenya, Mount Kilimanjaro), subtropical (Mount Mulanje, Manica Highlands) and warm temperate (northern Afrotemperate, southern Afrotemperate) clustering (Fig. 2). On a higher level, the warm temperate forests are distinct from the northern forests.

**Table 1.** Number of tree species, genera and families with the percentage of endemic species for six Afromontane forests from Mount Kenya to southern Afrotemperate forests.

	Southern Afrotemperate (34º S)	Northern Afrotemperate (24º S)	Manica Highlands (19º S)	Mount Mulanje (15º S)	Mount Kilimanjaro (3º S)	Mount Kenya (0º)
Number of species	90	129	181	156	176	165
Number of genera	72	96	135	131	134	129
Number of families	43	54	62	61	62	60
Number of endemic species^a^	25	16	14	14	11	8
Percentage of endemic species	27.78	12.4	7.74	8.97	6.25	4.85

^a^Number of species that occurred only in corresponding forest region in relation to all other forests in the dataset.

### Flower colour, size and pollination syndrome

**Fig. 3.**
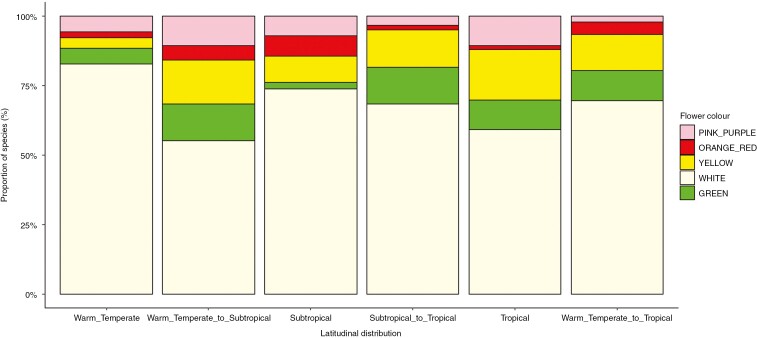
Proportional similarities of flower colours of Afromontane forest tree species for different latitudinal distribution categories: tropical (Mount Kenya and/or Mount Kilimanjaro only); subtropical (Mount Mulanje and/or Manica Highlands only); warm temperate (northern Afrotemperate and/or southern Afrotemperate only); warm temperate to tropical (species across whole range); subtropical to tropical (species limited to subtropical and tropical forests); and warm temperate to subtropical (species limited to warm temperate and subtropical forests).

**Fig. 4.**
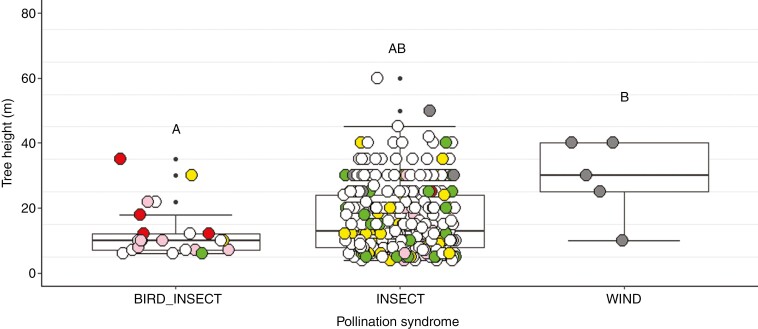
Relation between tree height (m) and pollination syndrome for Afromontane forest tree species at six forest regions between the southern Cape and Mount Kenya. Shared letters above plots indicate statistically similar means. Coloured points relate to flower colour: pink = pink-purple; red = orange-red; yellow = yellow; white = white; grey = no external flower.

Latitude had no relation to flower colour, flower size or pollination syndrome (Table 2). Flower colour proportions were statistically similar between all six latitudinal distribution categories, the dominant flower colour across forest regions being white (*n* = 207), followed by yellow (*n* = 38) and green (*n* = 29; Fig. 3). These colours represented 90.13 % (*n* = 274) of all flowering species, compared with 6.58 % (*n* = 20) for pink-purple and 3.29 % (*n* = 10) for orange-red flowers. The most dominant pollination syndrome was insect (91.35 %; *n* = 285), followed by bird–insect (5.77 %; *n* = 18), wind (1.6 %; *n* = 5) and bird (1.28 %; *n* = 4) pollination. A significant relation was found between tree height and pollination syndrome: bird–insect-pollinated trees were rarely taller than 22 m, whereas insect-pollinated trees encompassed the full spectrum of tree height, i.e. 4 up to 60 m. Wind-pollinated trees, although represented by five species only, were largely >25 m (Table 2; Fig. 4).

**Table 2.** Summative results of the GLMMs and LMMs showing the relation between reproductive traits of Afromontane forest trees and the variables latitudinal distribution, tree height and biogeographical affinity.

Response factor	Model variable	Akaike information criterion	*χ* ^2^	Degrees of freedom	*P* value
Flower colour (GLMM)	Latitudinal distribution	190.6	6.55	5	0.26
	Tree height	192.57	0.53	1	0.47
	Biogeographical affinity	191.09	1.05	2	0.59
Flower size (LMM)	Latitudinal distribution	802.66	5.06	5	0.41
	Tree height	809.84	4.24	1	0.04*
	Biogeographical affinity	804.81	1.21	2	0.55
Pollination syndrome (GLMM)	Latitudinal distribution	104.2	2.32	5	0.8
	Tree height	118.89	9	1	0.003**
	Biogeographical affinity	106.33	0	2	1
Fruit colour (GLMM)	Latitudinal distribution	283.14	5.1	5	0.4
	Tree height	287.13	1.08	1	0.3
	Biogeographical affinity	293.77	9.72	2	0.007**
Fruit size (LMM)	Latitudinal distribution	625.41	1.36	5	0.93
	Tree height	644.32	12.27	1	<0.001***
	Biogeographical affinity	631.77	1.72	2	0.42
Dispersal syndrome (GLMM)	Latitudinal distribution	205.24	4.41	5	0.49
	Tree height	209.36	0.53	1	0.47
	Biogeographical affinity	212.86	6.03	2	0.04*

### Fruit colour, size and dispersal syndromes

**Fig. 5.**
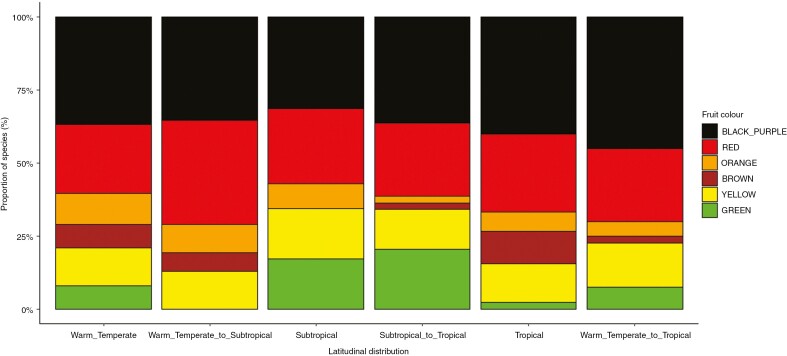
Proportional similarities of fruit colours for Afromontane forest tree species for the different latitudinal distribution categories: tropical (Mount Kenya and/or Mount Kilimanjaro only); subtropical (Mount Mulanje and/or Manica Highlands only); warm temperate (northern Afrotemperate and/or southern Afrotemperate only); warm temperate to tropical (species across whole range); subtropical to tropical (species limited to subtropical and tropical forests); and warm temperate to subtropical (species limited to warm temperate and subtropical forests).

**Fig. 6.**
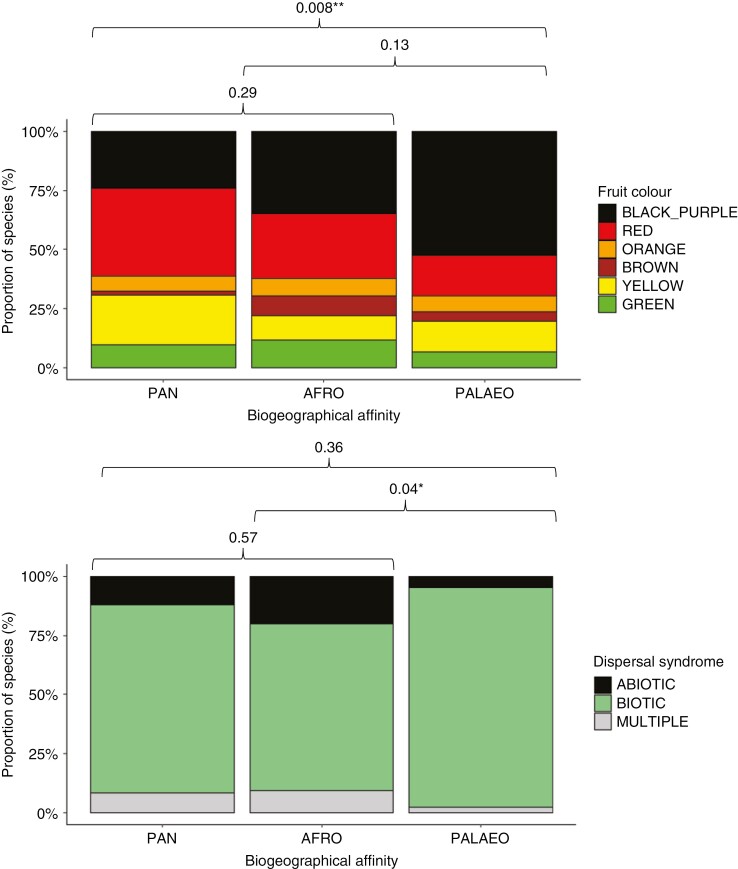
Proportions of fruit colour (A) and dispersal syndrome (B) of Afromontane tree species grouped into pantropical (Pan), Afrotropical (Afro) and palaeotropical (Palaeo) biogeographical affinities. Results of GLMMs are reported as *P*-values above the bars. *P <0.05; ** *P* <0.01.

**Fig. 7.**
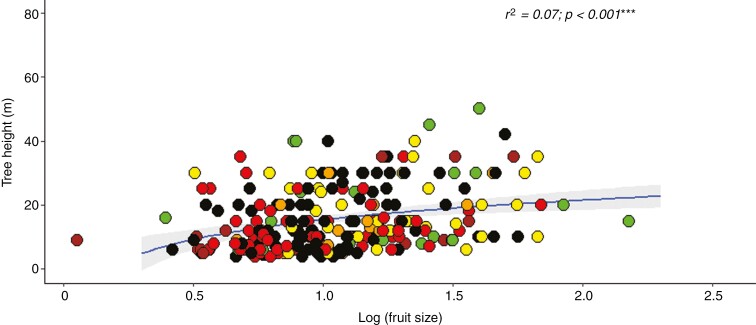
Relation between tree height (m) and log(fruit size) for Afromontane forest tree species between Mount Kenya (0º) and southern Afrotemperate forest (34º) regions. The blue line shows a logarithmic regression and the grey area depicts the 95 % confidence interval. Coloured points relate to fruit colour: black = black-purple; red = red; yellow = yellow; brown = brown; green = green; orange = orange.

Latitude had no relation to fleshy fruit colour, fruit size or dispersal syndrome (Table 2). Fruit colour proportions were statistically similar between all six latitudinal distribution categories, the dominant fruit colour being black-purple (*n* = 88; 37.77 %), followed by red (*n* = 62; 26.61 %) (Fig. 5). Cryptic colours, i.e. brown (*n* = 12), green (*n* = 22) and yellow (*n* = 33), were found for 28.76 % of species (*n* = 67), with only 6.87 % of species bearing orange fruit (*n* = 16). The most dominant dispersal syndrome was biotic (79.29 %; *n* = 245), followed by abiotic (13.59 %; *n* = 42) and multiple (7.12 %; *n* = 22). Fruit colour and dispersal syndrome revealed significant relations with biogeographical affinity (Table 2; Fig. 6). The distribution of fruit colour proportions differed between palaeotropical and pantropical genera, with palaeotropical genera revealing a relative increase in contrasting fruit colours driven by black-purple fruits and a relative decrease in cryptic fruit colours (Fig. 6A). Afrotropical genera showed lower than expected biotic dispersal compared with palaeotropical genera, with higher than expected abiotic dispersal (Fig. 6B). A significant relation was found between tree height and fruit size, taller trees correlating with larger fruits (Table 2; Fig. 7).

## DISCUSSION

Afromontane tree taxonomic diversity decreases from the tropics and subtropics towards southern latitudes, whereas southern Afrotemperate forests contain the highest proportion of endemic species. No effect of latitude was found on the tree reproductive traits tested here. We reject our hypothesis that flower and fruit colour of Afromontane forest trees decrease in morphological disparity away from tropical latitudes, with latitude having no relation to either flower or fruit colour. Fruit colour, interestingly, related to biogeographical affinity, with palaeotropical genera revealing a relative increase in contrasting fruit colours (black-purple, red) and a relative decrease in cryptic fruit colours, compared with pantropical genera. We reject our hypothesis that flower and fruit size decrease away from tropical latitudes; however, tree height showed positive correlations with fruit size regardless of latitude. Lastly, we reject our hypothesis that wind pollination and abiotic dispersal increase away from tropical latitudes; however, wind pollination was more prevalent in taller tree species (relative to bird pollination), and insect-pollinated trees encompassed the whole spectrum of tree height.

The diversity distribution of Afromontane tree species along the latitudinal gradient is consistent with global trends, with tropical latitudes hosting the highest levels of tree richness (Gatti *et al*., 2022). Forest area, interestingly, did not influence tree diversity patterns; the Manica Highlands’ Afromontane forests, at ~6000 ha in extent, had more tree species than the largest Afromontane forest included here, at Mount Kenya (~160 000 ha). The reported latitudinal pattern is contrasted to an inverse pattern in Africa: high plant diversity of the temperate Cape Floristic Region *sensu lato*, which does not conform to global latitudinal trends (Linder, 2014). Afromontane forest flora, importantly, is largely of tropical origin (White, 1978; Linder, 2014), which since the Oligocene–Miocene expanded and contracted across the continent in relation to fluctuating climatic conditions (Migliore *et al*., 2020; Couvreur *et al*., 2021). The uplifting of the East African Plateau throughout the mid-Miocene saw the formation of migration routes linking north and south Africa (Couvreur *et al*., 2021). As noted by Migliore *et al*. (2020), research on Afromontane trees’ dispersal and migration history among currently isolated Afromontane areas is scant; however, recent phylogeographical work on *Prunus africana* (Kadu *et al*., 2011) and *Podocarpus latifolius* (Migliore *et al*., 2020), both widespread Afromontane forest trees, indicate a north-to-south migration route. The arid Limpopo River valley, as shown by Lawes *et al*. (2007), separates the Manica Highlands from northern Afrotemperate forests, and provides a significant barrier to tropical elements dispersing southward. Our data suggest a sharp decrease in tree taxonomic diversity for Afromontane forests south of the Manica Highlands. Further south, the semi-arid Bedford gap, separating forests of the eastern and south-eastern Cape (Lawes, 1990), could explain the further decrease in species richness towards southern Afrotemperate forests.

The higher endemism of Afromontane forest trees shown for southern Afrotemperate forests could be argued to be a result of the age of the substrate (>300 compared with <6 mya for Mount Kilimanjaro and Mount Kenya) and allowing more time for speciation (Schluter, 2016). This would not explain the relatively lower levels of endemism for other, equally older substrates on which Afromontane forests are found, for instance the Manica Highlands (>300 mya) or Mount Mulanje (>130 mya). Southern Afrotemperate forests, in part due to their southerly location, could have seen longer periods of isolation from northern Afromontane forests (Lawes *et al*., 2007), allowing more time for speciation. This leads to the question whether pollinator shifts could have facilitated sympatric speciation. Another explanation could lie in its nestedness in the diverse Austro-temperate clade and proximity to coastal thicket floras (Linder *et al*., 2005; Linder, 2014); Afromontane floras, as pointed out by Linder *et al*. (2005), are often enriched by surrounding floral elements, or ecological transgressors (White, 1978). These considerations, along with a temperate location and climate, could explain why the warm temperate forest regions are relatively unique among the Afromontane forests studied here.

We suggest three possible hypotheses explaining the results pertaining to latitudinal similarities in tree reproductive traits. Firstly, the latitudinal similarities reported are indicative of phylogenetic conservatism. Secondly, there is no change in selective pressures across the latitudinal gradient, with similar mutualistic partners, thus indicating convergence. Thirdly, selective forces of plant mutualists on tree reproductive traits remain proportionately similar, regardless of whether diversity decreases away from tropical latitudes.

### First hypothesis: phylogenetic conservatism

Phylogenetic conservatism, in which legacy traits of ancestors persist (Prinzing *et al*., 2001), is supported both by the lack of latitudinal patterns on most of the traits tested here, and traits relating to biogeographical affinity (fruit colour, dispersal syndrome). From an analysis of 9370 plant species across a broad taxonomic range in China, occurring in tropical, subtropical and temperate regions, phylogenetic conservatism explained the majority of fruit trait variation, with little variation explained by climate or growth form (Wang *et al*., 2022). Evolutionary rates of plants, especially trees, are slow (Herrera, 1987). This has led many authors to suggest that, rather than the intuitive link between frugivore selection pressure and fruit traits, non-adaptive processes (i.e. phylogenetic inertia) might be shaping current fruit trait distribution (Valenta and Nevo, 2020). For the historically expanding and contracting Afromontane tree flora, underlying fruit traits could drive plant distribution patterns; thus, phylogenetic niche conservatism could explain the reported similarities. Previous work on coastal, scarp and Afrotemperate forests in South Africa showed how tree reproductive traits are highly phylogenetically conserved, which included flower colour, flower size, pollination syndrome, fruit size and the fruit colours black-purple, red, brown and yellow (Griffiths and Lawes, 2006). Although we did not specifically test for phylogenetic conservatism, we suggest that it is a viable hypothesis explaining the reported patterns.

### Second hypothesis: convergence

Divergence in reproductive trait expression across geographic zones often results from morphological, taxonomical or behavioural differences in plant mutualists and related selective pressures. Alternatively, plant communities may converge when pollinator and frugivore selective forces are similar, or even shared (Newman *et al*., 2014). For Afromontane tree flora, reproductive traits are argued to be ancient, predating current species’ distribution, having converged towards similar, and historically connected, biotic selective forces. This becomes especially plausible when considering current, and arguably divergent, biotic and abiotic selective pressures between temperate and tropical latitudes. A much expanded and connected Afromontane flora, at lower altitudes, has been suggested by Assèdé *et al*. (2021), Migliore *et al*. (2020, 2022) and Allen *et al*. (2021) in response to palaeoclimatic fluctuations. The similarities in reproductive traits of Afromontane trees, despite the isolation between study areas, lends further credence to the connection theory, in which the isolation of forests along the Afromontane archipelago are, in geological terms, fairly recent (Raven and Axelrod, 1974; Lawes, 1990; Kadu *et al*., 2011). During the interglacial to preglacial period, 100 000–18 000 years before present, Afromontane forests would have reached a wider area distribution than the present distribution, which decreased in extent (albeit fluctuating) since the last glacial maximum (18 000 years before present; Lawes, 1990). During palaeohistoric periods of forest expansion, forest fauna was able to disperse between presently highly isolated forests (Lawes, 1990). This is seen in the fragmented distribution of numerous endemic forest frugivores, e.g. the Samango monkey (*Cercopithecus mitis*) (Lawes, 1990), Cape parrot (*Poicephalus robustus*) (Downs *et al*., 2014) and Knysna turaco (*Tauraco corythaix*) (Perktaş *et al*., 2020). Thus, the similarity in pollinator and frugivore morphology, taxonomy and behaviour in now isolated forests could drive trait convergence despite latitudinal differences in climate and diversity.

### Third hypothesis: similar proportional selective forces

Despite biodiversity decreasing away from lower latitudes, similar proportional selection pressures could exist across the continent. Previous work has shown how selection pressures differ across latitudinal gradients, exerting intraspecific variation in plant traits (Sobral *et al*., 2013, 2015). Our methodology does not allow comparisons between populations of single species, but rather presents interspecific, population-level differences in reproductive trait composition. Although there is a change in diversity of mutualistic partners with latitude, the proportions of functional groups (birds, insects, mammals) to tree diversity remain, in broad terms, constant. For example, bird species richness in southern Afrotemperate forests numbers 35 (Koen, 1992), compared with Afromontane forests in KwaZulu–Natal, South Africa (29º S), which had a bird richness of 61 (Wethered and Lawes, 2003). In an Afromontane forest at Mount Kenya, a bird richness of 70 was recorded (Mahiga *et al*., 2019). Proportionately, these increases are closely mimicked in tree richness reported here. Arguably, this leads to little change in the proportion of reproductive traits, explaining trait similarity.

From southern temperate, Andean forests, latitude did not correlate with pollination or dispersal syndromes of plants (Aizen and Ezcurra, 1998). Rather, longitude, linked to a rainfall gradient from west to east, explained changes in plant–animal mutualisms (Aizen and Ezcurra, 1998). Although not tested here, longitude could explain plant reproductive traits, especially for South African Afromontane forests, which show great west-to-east variation in elevation, ocean temperatures, neighbouring vegetation types and tree species richness (Cowling *et al*., 1997). The patterns reported here should be viewed as broad-scale trends, both spatially and morphologically. Investigations into finer-scale variables, both inter- and intraspecific, e.g. specific pollinator identity, flower shape or petal reflectance, may reveal latitudinal effects not shown here. However, we present three important considerations with regard to the Afromontane tree flora: (1) evolutionary selective forces, i.e. biogeographical affinity, is deemed important for dispersal syndrome and fruit colour; (2) environmental selective forces, i.e. tree height, is deemed important for pollination syndrome and fruit size; and (3) latitudinal selective forces exert little pressure on tree reproductive traits.

## Conclusions

Afromontane forests occur over a large latitudinal range with differential abiotic, and perhaps biotic, selection pressures. The decrease in tree taxonomic diversity shown from tropical and subtropical to temperate latitudes becomes apparent south of the Manica Highlands. Southern Afrotemperate forests stand out as having the most endemic species relative to the forest regions included here. Despite these considerations, trends in reproductive traits of Afromontane forest trees show no relation to latitude. We propose that reproductive trait similarity across latitude can be explained by phylogenetic conservatism, trait convergence or similar proportional selective forces between temperate and tropical latitudes. We suggest that tree reproductive traits in this system are ancient and persist throughout significant palaeohistoric shifts in climate and a once greater extent of Afromontane flora across the African continent.

## SUPPLEMENTARY DATA

Supplementary data are available online at https://academic.oup.com/aob and consist of the following. Table S1: summary of the six Afromontane forest regions included in the study, including respective approximate area of forest, elevational range, annual rainfall range, substrate type, estimated age of mountain and the number of tree species. Table S2: tree species list with references used to gather data on genus distribution, flower and fruit colour, flower and fruit size and pollination and dispersal syndrome. Table S3: Summary of 331 tree species occurring in six Afromontane forest regions from the southern Cape to Mount Kenya, including their respective heights, latitudinal distributions and biogeographical affinities, as well as reproductive traits, ordered alphabetically by species name..

mcad080_suppl_Supplementary_Table_S1

mcad080_suppl_Supplementary_Table_S2

mcad080_suppl_Supplementary_Data
