# Supplementary material for: Weak latitudinal trends in reproductive traits of Afromontane forest trees
Source: Ann Bot. 2023 Jul 5;133(5-6):711–24. doi: 10.1093/aob/mcad080 (PMC11082511; doi:10.1093/aob/mcad080)
Supplement: mcad080_suppl_Supplementary_Table_S1 [file mcad080_suppl_supplementary_table_s1.docx]

**Supplementary data Table S1:** Summary of the six Afromontane forest regions included in the study, including respective approximate area of forest, elevational range, annual rainfall range, substrate type, estimated age of mountain and the number of tree species.

| **Afromontane forest** | **Area** | **Elevation** | **Annual rainfall** | **Substrate** | **Age of mountain** | **Tree species** |
| --- | --- | --- | --- | --- | --- | --- |
| Southern Afrotemperate (34º S) | 74 848 ha | 10 - 1060 masl | 863 mm | Quartsitic sandstone | >300 mya | 90 |
| Northern Afrotemperate (24º S) | 38 095 ha | 1100 - 2000 masl | 1042 mm | Quartsitic sandstone and rarely volcanic rocks | >300 mya | 129 |
| Manica Highlands (19º S) | 6 120 ha | 1650 - 2100 masl | 2000 - 3000 mm | Quartzites, schists, limestone | >400 mya | 181 |
| Mount Mulanje (15º S) | 4 600 ha | 1600 - 1950 masl | 2425 - 3108 mm | Coalescing syenite, quartz syenite and granite intrusions | 130 mya | 156 |
| Mount Kilimanjaro (3º S) | 100 000 ha | 1600 - 3100 masl | 1900 - 2700 mm | Volcanic deposits | <4 mya | 176 |
| Mount Kenya (0º) | 165 000 ha | 1450 - 3890 masl | 870 - 2170 mm | Volcanic deposits | <6 mya | 165 |
