# Supplementary material for: Weak latitudinal trends in reproductive traits of Afromontane forest trees
Source: Ann Bot. 2023 Jul 5;133(5-6):711–24. doi: 10.1093/aob/mcad080 (PMC11082511; doi:10.1093/aob/mcad080)
Supplement: mcad080_suppl_Supplementary_Table_S2 [file mcad080_suppl_supplementary_table_s2.docx]

**Supplementary data Table S2:** Tree species list with references used to gather data on genus distribution, flower and fruit colour, flower and fruit size and pollination and dispersal syndrome. Plant of the World Online, Royal Botanical Gardens KEW, was used as taxonomical framework.

| **Tree number** | **Genus** | **Species** | **Reference/s** |
| --- | --- | --- | --- |
| *1.* | *Acokanthera* | *oppositifolia* | Govaerts et al (2000); Coates Palgrave (2002) |
| *2.* | *Acokanthera* | *schimperi* | Govaerts et al (2000); Omino (2002) |
| *3.* | *Adenocarpus* | *mannii* | White et al. (2001) |
| *4.* | *Afrocanthium* | *mundianum* | White et al. (2001); Venter (2011) |
| *5.* | *Afrocarpus* | *falcatus* | Knopf et al. (2012); Venter (2011) |
| *6.* | *Agarista* | *salicifolia* | White et al. (2001) |
| *7.* | *Aidia* | *micrantha* | White et al. (2001); Hyde et al. (2020); Timberlake et al. (2021) |
| *8.* | *Alangium* | *chinense* | White et al. (2001); Hyde et al. (2020); Timberlake et al. (2021) |
| *9.* | *Albizia* | *gummifera* | White et al. (2001); Hyde et al. (2020); Timberlake et al. (2021) |
| *10.* | *Albizia* | *schimperiana* | White et al. (2001); Hyde et al. (2020); Timberlake et al. (2021) |
| *11.* | *Alchornea* | *hirtella* | White et al. (2001); Bingham et al. (2021) |
| *12.* | *Allophylus* | *abyssinicus* | White et al. (2001); Coates Palgrave (2002) |
| *13.* | *Allophylus* | *chaunostachys* | White et al. (2001); Hyde et al. (2020); Timberlake et al. (2021) |
| *14.* | *Allophylus* | *chirindensis* | White et al. (2001); Hyde et al. (2020); Timberlake et al. (2021); Davies & Verdcourt (1998) |
| *15.* | *Allophylus* | *decipiens* | White et al. (2001); Venter (2011) |
| *16.* | *Allophylus* | *ferrugineus* | White et al. (2001); Davies & Verdcourt (1998) |
| *17.* | *Aningeria* | *adolfi-friedericii* | White et al. (2001); Lemmens (2007); Lemmens (2021) |
| *18.* | *Anthocleista* | *grandiflora* | White et al. (2001); Hyde et al. (2020); Timberlake et al. (2021) |
| *19.* | *Antidesma* | *vogelianum* | White et al. (2001); Hyde et al. (2020); Timberlake et al. (2021) |
| *20.* | *Aphloia* | *theiformis* | White et al. (2001); Kaiser-Bunbery et al. (2009); Hyde et al. (2020); Timberlake et al. (2021) |
| *21.* | *Apodytes* | *dimidiata* | White et al. (2001); Hyde et al. (2020); Timberlake et al. (2021) |
| *22.* | *Astropanax* | *goetzenii* | White et al. (2001); Hyde et al. (2020); Timberlake et al. (2021); Cannon (1978) |
| *23.* | *Astropanax* | *polysciadus* | White et al. (2001); Cannon (1978); Thjis (2014) |
| *24.* | *Bersama* | *abyssinica* | White et al. (2001); Hyde et al. (2020); Timberlake et al. (2021) |
| *25.* | *Bersama* | *swynnertonii* | White et al. (2001); Hyde et al. (2020); Timberlake et al. (2021); White 1(1966) |
| *26.* | *Bersama* | *tysoniana* | White et al. (2001); Boon ((2010)) |
| *27.* | *Brachylaena* | *glabra* | Beentje (2000) |
| *28.* | *Brachylaena* | *huillensis* | Beentje (2000) |
| *29.* | *Brachylaena* | *neriifolia* | Beentje (2000); Venter (2011) |
| *30.* | *Bridelia* | *micrantha* | White et al. (2001); Coates Palgrave (2002) |
| *31.* | *Bridelia* | *taitensis* | White et al. (2001); Smith (1987); Thjis (2014) |
| *32.* | *Brillantaisia* | *cicatricosa* | White et al. (2001); Hyde et al. (2020); Timberlake et al. (2021) |
| *33.* | *Brucea* | *antidysenterica* | White et al. (2001); Burkhill (2000) |
| *34.* | *Buddleja* | *auriculata* | White et al. (2001); Coates Palgrave (2002) |
| *35.* | *Buddleja* | *pulchella* | White et al. (2001); Coates Palgrave (2002) |
| *36.* | *Buddleja* | *saligna* | White et al. (2001); Venter (2011) |
| *37.* | *Buddleja* | *salviifolia* | White et al. (2001); Venter (2011) |
| *38.* | *Burchellia* | *bubalina* | Venter (2011); Geldenhuys (1996) |
| *39.* | *Buxus* | *macowanii* | White et al. (2001); Pooley (1993); Geldenhuys (1996) |
| *40.* | *Buxus* | *nyasica* | White et al. (2001); Baker (1913) |
| *41.* | *Calodendrum* | *capense* | White et al. (2001); Venter (2011) |
| *42.* | *Calpurnia* | *aurea subsp. aurea* | Beaumont et al. (1999); Hyde et al. (2020); Timberlake et al. (2021); Coates Palgrave (2002) |
| *43.* | *Canthium* | *bugoyensis* | White et al. (2001); Bridson (1998) |
| *44.* | *Canthium* | *ciliatum* | White et al. (2001); Coates Palgrave (2002) |
| *45.* | *Canthium* | *inerme* | White et al. (2001); Venter (2011) |
| *46.* | *Canthium* | *kuntzeanum* | White et al. (2001); Hyde et al. (2020); Timberlake et al. (2021) |
| *47.* | *Canthium* | *oligocarpum* | White et al. (2001); Hyde et al. (2020); Timberlake et al. (2021) |
| *48.* | *Carvalhoa* | *campanulata* | White et al. (2001) |
| *49.* | *Casearia* | *battiscombei* | White et al. (2001); Hyde et al. (2020); Timberlake et al. (2021) |
| *50.* | *Cassine* | *peragua* | White et al. (2001); Venter (2011) |
| *51.* | *Cassinopsis* | *ilicifolia* | Potgieter & Van Wyk (1994); Venter (2011) |
| *52.* | *Cassinopsis* | *tinifolia* | Potgieter & Van Wyk (1994); Hyde et al. (2020); Timberlake et al. (2021); Mendes (1963) |
| *53.* | *Cassipourea* | *gummiflua* | Potgieter & Van Wyk (1994); Hyde et al. (2020); Timberlake et al. (2021); White et al. (2001) |
| *54.* | *Cassipourea* | *malosana* | Potgieter & Van Wyk (1994); Hyde et al. (2020); Timberlake et al. (2021); White et al. (2001); Friis 1993 |
| *55.* | *Catha* | *edulis* | White et al. (2001); Hyde et al. (2020); Timberlake et al. (2021); Pooley (1993); Zhang et al. (2014) |
| *56.* | *Celtis* | *africana* | White et al. (2001); Venter (2011) |
| *57.* | *Chassalia* | *parvifolia* | White et al. (2001); Verdcourt (1989) |
| *58.* | *Clausena* | *anisata* | White et al. (2001); Hyde et al. (2020); Timberlake et al. (2021); Venter (2011) |
| *59.* | *Clerodendrum* | *johnstonii* | White et al. (2001); Fernandes et al. (2005) |
| *60.* | *Clutia* | *abyssinica* | White et al. (2001); Hyde et al. (2020); Timberlake et al. (2021); Ssali et al. (2018) |
| *61.* | *Clutia* | *pulchella* | White et al. (2001); Venter (2011); Thomson & Edwards (2001) |
| *62.* | *Coffea* | *ligustroides* | White et al. (2001); Davis et al. (2006) |
| *63.* | *Coffea* | *salvatrix* | White et al. (2001); Hyde et al. (2020); Timberlake et al. (2021) |
| *64.* | *Cola* | *greenwayi* | White et al. (2001); Hyde et al. (2020); Timberlake et al. (2021); Coates Palgrave (2002) |
| *65.* | *Combretum* | *kraussii* | White et al. (2001): Carr (1988) |
| *66.* | *Cornus* | *volkensii* | White et al. (2001); Hyde et al. (2020); Timberlake et al. (2021) |
| *67.* | *Craibia* | *brevicaudata subs. baptistarum* | White et al. (2001); Hyde et al. (2020); Timberlake et al. (2021); Brink (2006) |
| *68.* | *Crotalaria* | *capensis* | White et al. (2001); Pooley (1993); Le Roux et al. (2011) |
| *69.* | *Croton* | *macrostachyus* | White et al. (2001); Burkhill (1994) |
| *70.* | *Croton* | *megalocarpus* | White et al. (2001); Hyde et al. (2020); Timberlake et al. (2021); Burkhill (1994) |
| *71.* | *Croton* | *sylvaticus* | White et al. (2001); Coates Palgrave (2002) |
| *72.* | *Cryptocarya* | *liebertiana* | White et al. (2001); Hyde et al. (2020); Timberlake et al. (2021) |
| *73.* | *Cryptocarya* | *woodii* | White et al. (2001); Pooley (1993) |
| *74.* | *Cunonia* | *capensis* | Bradford et al. (2004); Venter (2011) |
| *75.* | *Curtisia* | *dentata* | Chapman (2008); Venter (2011) |
| *76.* | *Cussonia* | *holstii* | White et al. (2001); Thulin et al. (2008) |
| *77.* | *Cussonia* | *spicata* | White et al. (2001); Hyde et al. (2020); Timberlake et al. (2021) |
| *78.* | *Cylicomorpha* | *parviflora* | White et al. (2001); Hemsley (1958); Kupicha (1978) |
| *79.* | *Dais* | *cotinifolia* | White et al. (2001); Van Wyk & Van Wyk (1997) |
| *80.* | *Dasylepis* | *integra* | Sleumer (1975); Lovett et al. (1993); Lovett et al. (2003) |
| *81.* | *Deinbollia* | *kilimandscharica* | White et al. (2001); Davies & Verdcourt (1998) |
| *82.* | *Dicranolepis* | *usambarica* | Peterson (1978); Thjis (2014) |
| *83.* | *Didymosalpinx* | *norae* | Robbrecht & De Block (2002); Hyde et al. (2020); Timberlake et al. (2021) |
| *84.* | *Diospyros* | *abyssinica* | White et al. (2001); Hyde et al. (2020); Timberlake et al. (2021) |
| *85.* | *Diospyros* | *dichrophylla* | White et al. (2001); Venter (2011) |
| *86.* | *Diospyros* | *whyteana* | White et al. (2001); Venter (2011) |
| *87.* | *Discopodium* | *penninervium* | White et al. (2001); Wright (1905); Gonçalves (2005) |
| *88.* | *Dombeya* | *burgessiae* | White et al. (2001); Verdoorn & Herman (1986); Hyde et al. (2020); Timberlake et al. (2021) |
| *89.* | *Dombeya* | *torrida* | White et al. (2001); Cheek & Dorr (2007); Brink (2007) |
| *90.* | *Dovyalis* | *abyssinica* | White et al. (2001); Thulin et al. (2008) |
| *91.* | *Dovyalis* | *lucida* | White et al. (2001); Hyde et al. (2020); Timberlake et al. (2021); Roux (2003) |
| *92.* | *Dovyalis* | *macrocalyx* | White et al. (2001); Hyde et al. (2020); Timberlake et al. (2021) |
| *93.* | *Dovyalis* | *rhamnoides* | White et al. (2001); Venter (2011) |
| *94.* | *Dovyalis* | *zeyheri* | White et al. (2001); Coates Palgrave (2002) |
| *95.* | *Drypetes* | *gerrardii* | White et al. (2001); Hyde et al. (2020); Timberlake et al. (2021) |
| *96.* | *Ehretia* | *cymosa subs. divaricata* | White et al. (2001); Verdcourt (1991); Burkill (1985) |
| *97.* | *Ekebergia* | *capensis* | White et al. (2001); Venter (2011) |
| *98.* | *Ekebergia* | *pterophylla* | White et al. (2001); Coates Palgrave (2002) |
| *99.* | *Elaeodendron* | *croceum* | Archer & Van Wyk (1998); Venter (2011) |
| *100.* | *Elaeodendron* | *schinoides* | White et al. (2001); Venter (2011) |
| *101.* | *Empogona* | *lanceolata* | White et al. (2001); Coates Palgrave (2002); Van Wyk & Van Wyk (1997) |
| *102.* | *Englerophytum* | *magalismontanum* | White et al. (2001); Coates Palgrave (2002); Hyde et al. (2020); Timberlake et al. (2021) |
| *103.* | *Entandrophragma* | *excelsum* | White et al. (2001); Styles & White (1991) |
| *104.* | *Erica* | *arborea* | White et al. (2001); Beentje (2000); Beentje (2005) |
| *105.* | *Erica* | *mannii* | White et al. (2001); Govaerts (2003) |
| *106.* | *Erica* | *nyassana* | White et al. (2001) |
| *107.* | *Erythrina* | *lysistemon* | White et al. (2001); Van Wyk & Van Wyk (1997); Coates Palgrave (2002) |
| *108.* | *Erythrococca* | *bongensis* | White et al. (2001); Smith (1987) |
| *109.* | *Erythrococca* | *polyandra* | White et al. (2001); Hyde et al. (2020); Timberlake et al. (2021) |
| *110.* | *Erythroxylum* | *emarginatum* | White et al. (2001); Hyde et al. (2020); Timberlake et al. (2021) |
| *111.* | *Euclea* | *crispa* | White et al. (2001); Coates Palgrave (2002) |
| *112.* | *Euclea* | *divinorum* | White et al. (2001); Van Wyk & Van Wyk (1997); Boon ((2010)) |
| *113.* | *Fagaropsis* | *angolensis* | White et al. (2001); Hyde et al. (2020); Timberlake et al. (2021); Bekele-Tesemma et al. (1993) |
| *114.* | *Faurea* | *arborea* | White et al. (2001); Rourke (1988); Brummitt & Marner (1993) |
| *115.* | *Faurea* | *macnaughtonii* | White et al. (2001); Rourke (1998); Von Breitenbach (1974) |
| *116.* | *Faurea* | *racemosa* | White et al. (2001); Rourke (1998); Marner (1989) |
| *117.* | *Faurea* | *rubriflora* | White et al. (2001); Rourke (1998); Hyde et al. (2020); Timberlake et al. (2021) |
| *118.* | *Fernandoa* | *abbreviata* | White et al. (2001); Hyde et al. (2020); Timberlake et al. (2021) |
| *119.* | *Ficus* | *burtt-davyi* | White et al. (2001); Boon (2010) |
| *120.* | *Ficus* | *chirindensis* | White et al. (2001); Hyde et al. (2020); Timberlake et al. (2021) |
| *121.* | *Ficus* | *craterostoma* | White et al. (2001); Boon (2010) |
| *122.* | *Ficus* | *scassellatii subs. scassellatii* | White et al. (2001); Hyde et al. (2020); Timberlake et al. (2021) |
| *123.* | *Ficus* | *sur* | White et al. (2001); Van Wyk & Van Wyk (1997); Coates Palgrave (2002) |
| *124.* | *Ficus* | *thonningii* | White et al. (2001); Berg & Hijman (1989) |
| *125.* | *Ficus* | *vallis-choudae* | White et al. (2001); Hyde et al. (2020); Timberlake et al. (2021) |
| *126.* | *Filicium* | *decipiens* | White et al. (2001); Davies & Verdcourt (1998) |
| *127.* | *Flacourtia* | *indica* | White et al. (2001); Thulin (1993) |
| *128.* | *Galiniera* | *saxifraga* | White et al. (2001) |
| *129.* | *Gambeya* | *gorungosana* | White et al. (2001); Hyde et al. (2020); Timberlake et al. (2021); Dominy & Duncan (2005); |
| *130.* | *Garcinia* | *kingaensis* | White et al. (2001); Hyde et al. (2020); Timberlake et al. (2021) |
| *131.* | *Garcinia* | *tanzaniensis* | White et al. (2001) |
| *132.* | *Garcinia* | *volkensii* | White et al. (2001); Robson (1961) |
| *133.* | *Gonioma* | *kamassi* | Govaerts (2003); Venter (2011) |
| *134.* | *Grewia* | *occidentalis* | White et al. (2001) Zietsman 1991; Venter (2011) Venter (2011) Venter (2011) |
| *135.* | *Grewia* | *similis* | White et al. (2001); Whitehouse et al. (2001) |
| *136.* | *Grewia* | *stolzii* | White et al. (2001); Hyde et al. (2020); Timberlake et al. (2021); Whitehouse et al. (2001) |
| *137.* | *Gymnosporia* | *harveyana* | Jordaan & Van Wyk (2006); Gunn & Codd (1981); Hyde et al. (2020); Timberlake et al. (2021) |
| *138.* | *Gymnosporia* | *nemorosa* | Jordaan & Van Wyk (2006); Van Wyk & Van Wyk (1997) |
| *139.* | *Hagenia* | *abyssinica* | White et al. (2001); Mendes (1978); Bekele-Tessema et al. (1993) |
| *140.* | *Halleria* | *lucida* | White et al. (2001) |
| *141.* | *Harpephyllum* | *caffrum* | Coates Palgrave (2002) |
| *142.* | *Harungana* | *madagascariensis* | White et al. (2001); Hyde et al. (2020); Timberlake et al. (2021) |
| *143.* | *Heinsenia* | *diervilleoides* | White et al. (2001); Hyde et al. (2020); Timberlake et al. (2021) |
| *144.* | *Heteromorpha* | *arborescens* | White et al. (2001); Hyde et al. (2020); Timberlake et al. (2021) |
| *145.* | *Hibiscus* | *burtt-davyi* | Pfeil & Crisp (2005); Hyde et al. (2020); Timberlake et al. (2021); Kudoh et al. (2006) |
| *146.* | *Hippobromus* | *pauciflorus* | Hyde et al. (2020); Timberlake et al. (2021); Venter (2011) |
| *147.* | *Homalium* | *dentatum* | White et al. (2001); Hyde et al. (2020); Timberlake et al. (2021); Roux (2003) |
| *148.* | *Hymenodictyon* | *floribundum* | White et al. (2001) Van Wyk & Van Wyk (1997); Hyde et al. (2020); Timberlake et al. (2021); Keay (1959) |
| *149.* | *Ilex* | *mitis* | White et al. (2001); Venter (2011) |
| *150.* | *Indigofera* | *lyallii subs lyallii* | White et al. (2001); Coates Palgrave (2002) |
| *151.* | *Itea* | *rhamnoides* | Jordaan (2012) |
| *152.* | *Ixora* | *scheffleri* | White et al. (2001); Njenga & Mugo (2020) |
| *153.* | *Juniperus* | *procera* | White et al. (2001); Hyde et al. (2020); Timberlake et al. (2021); Adams & Thornburg (2010) |
| *154.* | *Kiggelaria* | *africana* | White et al. (2001); Venter (2011) |
| *155.* | *Kuloa* | *usambarensis* | Beentje (1994); White et al. (2001) |
| *156.* | *Lachnostylis* | *hirta* | Dyer (1943); Venter (2011) |
| *157.* | *Lasianthus* | *kilimandscharicus subs. kilimandscharicus* | White et al. (2001); Hyde et al. (2020); Timberlake et al. (2021); Smedmark et al. (2014) |
| *158.* | *Lasiodiscus* | *usambarensis* | White et al. (2001); Hyde et al. (2020); Timberlake et al. (2021); Mwavu & Witkowski (2009) |
| *159.* | *Lasiosiphon* | *glauca* | White et al. (2001); Beentje (1994); Sasidharan et al. (1996) |
| *160.* | *Lauridia* | *tetragona* | Archer & Van Wyk (1997); Venter (2011) |
| *161.* | *Lepidotrichilia* | *volkensii* | White et al. (2001); Hyde et al. (2020); Timberlake et al. (2021) |
| *162.* | *Leptonychia* | *usambarensis* | White et al. (2001); Govaerts et al. (2021) |
| *163.* | *Loxostylis* | *alata* | Archer & Reynolds (2001) |
| *164.* | *Macaranga* | *capensis* | White et al. (2001); Hyde et al. (2020); Timberlake et al. (2021) |
| *165.* | *Macaranga* | *conglomerata* | White et al. (2001); Smith (1987) |
| *166.* | *Macaranga* | *mellifera* | White et al. (2001); Hyde et al. (2020); Timberlake et al. (2021) |
| *167.* | *Maerua* | *racemulosa* | Abreu et al. (2014); Venter (2011) |
| *168.* | *Maerua* | *triphylla johannis* | Abreu et al. (2014); Thulin (1993); Mollel (2013) |
| *169.* | *Maesa* | *lanceolata* | White et al. (2001); Hyde et al. (2020); Timberlake et al. (2021) |
| *170.* | *Mallotus* | *oppositifolius* | White et al. (2001); Mosango (2007); Hyde et al. (2020); Timberlake et al. (2021) |
| *171.* | *Manilkara* | *discolor* | White et al. (2001); Hyde et al. (2020); Timberlake et al. (2021) |
| *172.* | *Markhamia* | *lutea* | Prance (1975); Maroyi (2012) |
| *173.* | *Maytenus* | *acuminata* | White et al. (2001); Venter (2011) |
| *174.* | *Maytenus* | *albata* | White et al. (2001) |
| *175.* | *Maytenus* | *chasei* | White et al. (2001); Hyde et al. (2020); Timberlake et al. (2021) |
| *176.* | *Maytenus* | *peduncularis* | White et al. (2001); Venter (2011) |
| *177.* | *Maytenus* | *undata* | White et al. (2001); Boon (2010); Coates Palgrave (2002); Van Wyk & Van Wyk (1997) |
| *178.* | *Memecylon* | *natalense* | White et al. (2001); Van Wyk & Van Wyk (1997) |
| *179.* | *Mikaniopsis* | *bambuseti* | Beentje (2000) |
| *180.* | *Millettia* | *dura* | White et al. (2001); Orwa et al. (2009) |
| *181.* | *Millettia* | *oblata subs teitensis* | White et al. (2001); Thjis 2014 |
| *182.* | *Mitragyna* | *rubrostipulata* | White et al. (2001); Nyemb (2011) |
| *183.* | *Mussaenda* | *microdonta* | Verdcourt (1976) |
| *184.* | *Myrianthus* | *holstii* | White et al. (2001); Hyde et al. (2020); Timberlake et al. (2021) |
| *185.* | *Myrica* | *pilulifera* | Polhill & Verdcourt (2000); Hyde et al. (2020); Timberlake et al. (2021) |
| *186.* | *Myrsine* | *melanophloeos* | White et al. (2001); Venter (2011) |
| *187.* | *Mystroxylon* | *aethiopicum* | Robson (1966); Venter (2011) |
| *188.* | *Necepsia* | *castaneifolia subs. chirindica* | Radcliffe-Smith (1996) |
| *189.* | *Neoboutonia* | *macrocalyx* | White et al. (2001); Radcliffe-Smith (1996) |
| *190.* | *Neocussonia* | *umbellifera* | White et al. (2001); Hyde et al. (2020); Timberlake et al. (2021) |
| *191.* | *Newtonia* | *buchananii* | White et al. (2001); Hyde et al. (2020); Timberlake et al. (2021) |
| *192.* | *Nidorella* | *vernonioides* | Nesom (1990); Beentje (2000) |
| *193.* | *Noronhia* | *battiscombei* | White et al. (2001); Hyde et al. (2020); Timberlake et al. (2021) |
| *194.* | *Noronhia* | *foveolata* | White et al. (2001); Venter (2011); Von Breitenbach (1985) |
| *195.* | *Noronhia* | *mildbraedii* | White et al. (2001) African Plants Thjis 2014 |
| *196.* | *Nuxia* | *congesta* | White et al. (2001); Hyde et al. (2020); Timberlake et al. (2021) |
| *197.* | *Nuxia* | *floribunda* | White et al. (2001); Venter (2011); Geldenhuys (1997) |
| *198.* | *Obetia* | *radula* | Hyde et al. (2020); Timberlake et al. (2021); Friis (1989); Thjis (2014) |
| *199.* | *Ochna* | *arborea subs. arborea* | White et al. (2001); Venter (2011) |
| *200.* | *Ochna* | *arborea subs. oconnorii* | White et al. (2001); Hyde et al. (2020); Timberlake et al. (2021) |
| *201.* | *Ochna* | *holstii* | White et al. (2001); Hyde et al. (2020); Timberlake et al. (2021) |
| *202.* | *Ochna* | *insculpta* | White et al. (2001); Davies & Verdcourt (1998) |
| *203.* | *Ocotea* | *bullata* | White et al. (2001); Venter (2011) |
| *204.* | *Ocotea* | *kenyensis* | White et al. (2001); Hyde et al. (2020); Timberlake et al. (2021) |
| *205.* | *Olea* | *capensis subs. capensis* | Venter (2011) |
| *206.* | *Olea* | *capensis subs. macrocarpa* | White et al. (2001); Venter (2011) |
| *207.* | *Olea* | *europaea subs. cuspidata* | White et al. (2001); Venter (2011) |
| *208.* | *Olea* | *welwitschii* | White et al. (2001); Turril (1952) |
| *209.* | *Olinia* | *emarginata* | White et al. (2001); Boon (2010); Coates Palgrave (2002); Van Wyk & Van Wyk (1997) |
| *210.* | *Olinia* | *rochetiana* | White et al. (2001); Verdcourt (1975) |
| *211.* | *Olinia* | *vanguerioides* | White et al. (2001); Hyde et al. (2020); Timberlake et al. (2021); Verdcourt (1978); Sebola & Balkwill (2009) |
| *212.* | *Olinia* | *ventosa* | White et al. (2001); Venter (2011) |
| *213.* | *Osyris* | *compressa* | White et al. (2001); Venter (2011) |
| *214.* | *Oxyanthus* | *speciosus* | White et al. (2001); Hyde et al. (2020); Timberlake et al. (2021) |
| *215.* | *Pauridiantha* | *paucinervis* | White et al. (2001); Verdcourt (1976) |
| *216.* | *Pauridiantha* | *symplocoides* | White et al. (2001); Hyde et al. (2020); Timberlake et al. (2021); Verdcourt (1976) |
| *217.* | *Pavetta* | *abyssinica* | White et al. (2001); Verdcourt (1976) |
| *218.* | *Pavetta* | *comostyla subs. comostyla* | White et al. (2001); Hyde et al. (2020); Timberlake et al. (2021) |
| *219.* | *Pavetta* | *hymenophylla* | White et al. (2001); Verdcourt (1976); Bridson (1978) |
| *220.* | *Pavetta* | *lanceolata* | White et al. (2001); Bridson (2001) |
| *221.* | *Pavetta* | *umtalensis* | White et al. (2001); Hyde et al. (2020); Timberlake et al. (2021) |
| *222.* | *Peddiea* | *africana* | White et al. (2001); Hyde et al. (2020); Timberlake et al. (2021); Van Wyk & Van Wyk (1997) |
| *223.* | *Pistacia* | *aethiopica* | Kokwaro (1968); Thulin (1993) |
| *224.* | *Pittosporum* | *abyssinicum* | White et al. (2001); Cufodontis (1966) |
| *225.* | *Pittosporum* | *viridiflorum* | White et al. (2001); Venter (2011) |
| *226.* | *Platylophus* | *trifoliatus* | Venter (2011) |
| *227.* | *Podocarpus* | *henkelii* | White et al. (2001); Palmer & Pitman (1972) |
| *228.* | *Podocarpus* | *latifolius* | White et al. (2001); Venter (2011) |
| *229.* | *Polyscias* | *albersiana* | White et al. (2001); Tennant (1960) |
| *230.* | *Polyscias* | *fulva* | White et al. (2001); Hyde et al. (2020); Timberlake et al. (2021) |
| *231.* | *Polyscias* | *kikuyuensis* | White et al. (2001); Tennant (1960) |
| *232.* | *Polyscias* | *stuhlmannii* | White et al. (2001); Tennant (1960); Thijs (2014) |
| *233.* | *Premna* | *maxima* | Harley et al. (2004); Brink (2007) |
| *234.* | *Protorhus* | *longifolia* | Pell (2004); Van Wyk & Van Wyk (1997); Coates Palgrave (2002) |
| *235.* | *Prunus* | *africana* | White et al. (2001); Pooley (1993) |
| *236.* | *Psychotria* | *alsophila* | White et al. (2001); Verdcourt (1976) |
| *237.* | *Psychotria* | *capensis* | White et al. (2001); Hyde et al. (2020); Timberlake et al. (2021) |
| *238.* | *Psychotria* | *cyathicalyx* | White et al. (2001); Verdcourt (1976) |
| *239.* | *Psychotria* | *fractinervata* | White et al. (2001); Verdcourt (1976) |
| *240.* | *Psychotria* | *lauracea* | White et al. (2001); Verdcourt (1976); Thjis (2014) |
| *241.* | *Psychotria* | *mahonii* | White et al. (2001); Hyde et al. (2020); Timberlake et al. (2021) |
| *242.* | *Psychotria* | *orophila* | White et al. (2001); Verdcourt (1976) |
| *243.* | *Psychotria* | *zombamontana* | White et al. (2001); Hyde et al. (2020); Timberlake et al. (2021) |
| *244.* | *Psydrax* | *obovata subs. obovata* | Bridson (1985); Venter (2011) |
| *245.* | *Psydrax* | *parviflorus* | Bridson (1985); Bridson (1998) |
| *246.* | *Psydrax* | *schimperianus* | White et al. (2001); Bridson & Thulin (2006) |
| *247.* | *Ptaeroxylon* | *obliquum* | Hyde et al. (2020); Timberlake et al. (2021); Archer & Reynolds (2001) |
| *248.* | *Pterocelastrus* | *echinatus* | White et al. (2001); Hyde et al. (2020); Timberlake et al. (2021) |
| *249.* | *Pterocelastrus* | *rostratus* | White et al. (2001); Venter (2011) |
| *250.* | *Pterocelastrus* | *tricuspidatus* | White et al. (2001); Venter (2011) |
| *251.* | *Pyrostria* | *chapmanii* | White et al. (2001); Govaerts (2003) |
| *252.* | *Rauvolfia* | *caffra* | White et al. (2001); Hyde et al. (2020); Timberlake et al. (2021) |
| *253.* | *Rauvolfia* | *mannii* | White et al. (2001); Omino (2002); Thjis (2014) |
| *254.* | *Rawsonia* | *burtt-davyi* | White et al. (2001); Wild (1960) |
| *255.* | *Rawsonia* | *lucida* | White et al. (2001); Hyde et al. (2020); Timberlake et al. (2021) |
| *256.* | *Rhamnus* | *prinoides* | White et al. (2001); Venter (2011) |
| *257.* | *Rinorea* | *angustifolia* | White et al. (2001); Hyde et al. (2020); Timberlake et al. (2021) |
| *258.* | *Rinorea* | *convallarioides subs. convallarioides* | White et al. (2001); Munzinger & Pauly (2003); Van Velzen et al. (2015); Hyde et al. (2020); Timberlake et al. (2021); Tchouto et al. (2009) |
| *259.* | *Ritchiea* | *albersii* | White et al. (2001); Hyde et al. (2020); Timberlake et al. (2021); Thijs (2014) |
| *260.* | *Robsonodendron* | *eucleiforme* | Archer & Van Wyk (1997); Venter (2011) |
| *261.* | *Rothmannia* | *capensis* | White et al. (2001); Venter (2011) |
| *262.* | *Rothmannia* | *fischeri* | White et al. (2001); Hyde et al. (2020); Timberlake et al. (2021) |
| *263.* | *Rutidea* | *fuscescens* | White et al. (2001); Hyde et al. (2020); Timberlake et al. (2021); Bridson & Verdcourt (2003) |
| *264.* | *Rytigynia* | *adenodonta* | White et al. (2001); Bingham et al. (2021) |
| *265.* | *Rytigynia* | *eickii* | White et al. (2001); Bridson (1992); Thjis (2014) |
| *266.* | *Rytigynia* | *macrura* | White et al. (2001); Hyde et al. (2020); Timberlake et al. (2021) |
| *267.* | *Rytigynia* | *uhligii* | White et al. (2001); Hyde et al. (2020); Timberlake et al. (2021) |
| *268.* | *Schrebera* | *alata* | White et al. (2001); Van Wyk & Van Wyk (1997); Coates Palgrave (2002) |
| *269.* | *Scolopia* | *mundii* | White et al. (2001); Venter (2011) |
| *270.* | *Scolopia* | *oreophila* | White et al. (2001); Coates Palgrave (2002); Roux 2003 (JSTOR) |
| *271.* | *Scolopia* | *stolzii* | White et al. (2001); Hyde et al. (2020); Timberlake et al. (2021); Roux (2003) |
| *272.* | *Scolopia* | *zeyheri* | White et al. (2001); Venter (2011) |
| *273.* | *Scutia* | *myrtina* | White et al. (2001); Venter (2011) |
| *274.* | *Searsia* | *acuminatissima* | White et al. (2001); Fernandes & Fernandes (1996) |
| *275.* | *Searsia* | *chirindensis* | White et al. (2001); Moffett (2007); Venter (2011) |
| *276.* | *Searsia* | *dentata* | White et al. (2001); Moffett (2007); Hyde et al. (2020); Timberlake et al. (2021) |
| *277.* | *Searsia* | *lucida* | White et al. (2001); Moffett (2007); Venter (2011) |
| *278.* | *Searsia* | *natalensis* | White et al. (2001); Moffett (2007); Hyde et al. (2020); Timberlake et al. (2021) |
| *279.* | *Searsia* | *pyroides* | White et al. (2001); Moffett (2007); Hyde et al. (2020); Timberlake et al. (2021) |
| *280.* | *Searsia* | *tomentosa* | White et al. (2001); Moffett (2007); Hyde et al. (2020); Timberlake et al. (2021); Venter (2011) |
| *281.* | *Seemannaralia* | *gerrardii* | Foden & Potter (2005); Schmidt et al. (2002); Coates Palgrave (2002); Oskolski et al. (2010) |
| *282.* | *Sideroxylon* | *inerme* | Hyde et al. (2020); Timberlake et al. (2021); Venter (2011) |
| *283.* | *Solanecio* | *mannii* | Hyde et al. (2020); Timberlake et al. (2021); Beentje (2000); Chmel et al. (2021) |
| *284.* | *Solanum* | *phoxocarpum* | White et al. (2001); Vorontsova et al. (2010) |
| *285.* | *Spathodea* | *campanulata* | Bidgood et al. (2006); Rangaiah et al. (2004); Bosch (2002) |
| *286.* | *Strelitzia* | *alba* | Da Silva Vieira et al. (2012); Venter (2011) |
| *287.* | *Strelitzia* | *caudata* | Da Silva Vieira et al. (2012); Coates Palgrave (2002) |
| *288.* | *Strombosia* | *scheffleri* | White et al. (2001); Hyde et al. (2020); Timberlake et al. (2021) |
| *289.* | *Strychnos* | *decussata* | White et al. (2001); Van Wyk & Van Wyk (1997) |
| *290.* | *Strychnos* | *mellodora* | White et al. (2001); Hyde et al. (2020); Timberlake et al. (2021) |
| *291.* | *Strychnos* | *usambarensis* | White et al. (2001); Hyde et al. (2020); Timberlake et al. (2021) |
| *292.* | *Synsepalum* | *muelleri* | White et al. (2001); Hyde et al. (2020); Timberlake et al. (2021) |
| *293.* | *Syzygium* | *chimanimaniense* | Timberlake et al. (2021) |
| *294.* | *Syzygium* | *cordatum* | White et al. (2001); Van Wyk & Van Wyk (1997) |
| *295.* | *Syzygium* | *gerrardii* | White et al. (2001); Van Wyk & Van Wyk (1997) |
| *296.* | *Syzygium* | *guineense* | White et al. (2001); White (1978) |
| *297.* | *Syzygium* | *micklethwaitii* | White et al. (2001); Thjis (2014) |
| *298.* | *Tabernaemontana* | *stapfiana* | White et al. (2001); Hyde et al. (2020); Timberlake et al. (2021) |
| *299.* | *Tarchonanthus* | *littoralis* | Coates Palgrave (2002) (PZ); Herman (2002); Beentje (1999) |
| *300.* | *Tarchonanthus* | *trilobus* | Coates Palgrave (2002) (PZ); Herman (2002); Beentje (1999) |
| *301.* | *Trema* | *orientale* | White et al. (2001); Coates Palgrave (2002); Van Wyk & Van Wyk (1997) |
| *302.* | *Tricalysia* | *acokantheroides* | White et al. (2001) |
| *303.* | *Tricalysia* | *capensis* | White et al. (2001); Van Wyk (1984); Van Wyk & Van Wyk (1997); Robbrecht (1987) |
| *304.* | *Trichilia* | *dregeana* | White et al. (2001); Van Wyk & Van Wyk (1997) |
| *305.* | *Trichocladus* | *crinitus* | White et al. (2001); Venter (2011); Geldenhuys (1993) |
| *306.* | *Trichocladus* | *ellipticus* | White et al. (2001); Hyde et al. (2020); Timberlake et al. (2021); Geldenhuys (1993) |
| *307.* | *Trilepisium* | *madagascariense* | White et al. (2001); Hyde et al. (2020); Timberlake et al. (2021) |
| *308.* | *Trimeria* | *grandifolia* | Boon (2010); Coates Palgrave (2002); Venter (2011) |
| *309.* | *Turraea* | *abyssinica* | White et al. (2001); White & Styles (1963) |
| *310.* | *Turraea* | *holstii* | White et al. (2001); Hassan & Cheek (1999) |
| *311.* | *Turraea* | *robusta* | White et al. (2001); White (1991); Thjis (2014) |
| *312.* | *Vaccinium* | *exul* | White et al. (2001); Bester (2015) |
| *313.* | *Vachellia* | *abyssinica* | Thiele et al. (2011); Hyde et al. (2020); Timberlake et al. (2021); White et al. (2001) |
| *314.* | *Vangueria* | *apiculata* | White et al. (2001); Hyde et al. (2020); Timberlake et al. (2021) |
| *315.* | *Vangueria* | *volkensii* | White et al. (2001); Bridson (1998); Thjis (2014) |
| *316.* | *Vepris* | *bachmannii* | White et al. (2001); Hyde et al. (2020); Timberlake et al. (2021) |
| *317.* | *Vepris* | *lanceolata* | White et al. (2001); Venter (2011) |
| *318.* | *Vepris* | *nobilis* | White et al. (2001); Hyde et al. (2020); Timberlake et al. (2021) |
| *319.* | *Vepris* | *stolzii* | White et al. (2001); Kokwaro (1982) |
| *320.* | *Vernonia* | *syringifolia* | White et al. (2001); Beentje (2000); Bingham et al. (2021) |
| *321.* | *Virgilia* | *divaricata* | Venter (2011) |
| *322.* | *Virgilia* | *oroboides* | Venter (2011) |
| *323.* | *Warburgia* | *salutaris* | White et al. (2001); Coates Palgrave (2002); Palmer & Pitman (1972); Pooley (2003) |
| *324.* | *Warburgia* | *ugandensis* | White et al. (2001); Verdcourt (1958) |
| *325.* | *Widdringtonia* | *nodiflora* | White et al. (2001); Coates Palgrave (2002); Joffe (1993) |
| *326.* | *Widdringtonia* | *whytei* | White et al. (2001); Lewis (1960) |
| *327.* | *Xymalos* | *monospora* | White et al. (2001); Hyde et al. (2020); Timberlake et al. (2021) |
| *328.* | *Zanthoxylum* | *capense* | White et al. (2001); Venter (2011); Hyde et al. (2020); Timberlake et al. (2021) |
| *329.* | *Zanthoxylum* | *davyi* | White et al. (2001); Venter (2011); Hyde et al. (2020); Timberlake et al. (2021) |
| *330.* | *Zanthoxylum* | *gilletii* | White et al. (2001); Kokwaro (1982) |
| *331.* | *Zanthoxylum* | *usambarense* | White et al. (2001); Kokwaro (1982) |

**References**

1. Abreu, J. A., Martins, E. S., & Catarino, L. (2014). New species of *Maerua* (Capparaceae) from Angola. *Blumea-Biodiversity, Evolution and Biogeography of Plants*, *59* (1), 19-25.
2. Adams, R. P., & Thornburg, D. (2010). Seed dispersal in Juniperus: a review. Phytologia, 92 (3), 424-434.
3. Archer, R. H., & Van Wyk, A. E. (1997). A taxonomic revision of *Cassine* L. s. str. (Cassinoideae: Celastraceae). South African Journal of Botany, 63 (3), 146-157.
4. Archer, R. H., & Van Wyk, A. E. (1998). A taxonomic revision of *Elaeodendron* Jacq. (Cassinoideae: Celastraceae) in Africa. South African Journal of Botany, 64 (2), 93-109.
5. Archer, R., & Reynolds, Y. (2001) PlantzAfrica. http://pza.sanbi.org. South African National Biodiversity Institute, Pretoria, South Africa. Accessed on 16 February 2020.
6. Bachelier, J. B., & Endress, P. K. (2008). Floral structure of *Kirkia* (Kirkiaceae) and its position in Sapindales. Annals of Botany, 102 (4), 539-550.
7. Baker, J. G. (1913). Flora of Tropical Africa, Vol 6, Part 1, page 441 (with additions by C. H. Wright.) https://plants.jstor.org. Accessed on 17 April 2022.
8. Barnes, R. D., Filer, D. L., & Milton, S. J. (1996). *Acacia karroo*: monograph and annotated bibliography. Oxford Forestry Institute, University of Oxford.
9. Beaumont, A. J., Beckett, R. P., Edwards, T. J., & Stiron, C. H. (1999). Revision of the genus *Calpurnia* (Sophoreae: leguminosae). Bothalia, 29 (1), 5-23.
10. Beentje, H. J. (1999). The Genus *Tarchonanthus* (Compositae - Mutisieae). Kew Bulletin, 54 (1), 81–95. https://doi.org/10.2307/4111025
11. Beentje, H. J. (Ed.). (2000). Flora of Tropical East Africa - Compositae 1 (2000). Vol. 1. CRC Press.
12. Beentje, H.J. (1994). Kenya Trees, Shrubs and Lianas. National Museums of Kenya, Nairobi.
13. Bekele-Tesemma, A., & Tengnäs, B. (1993). Useful trees and shrubs of Ethiopia: identification, propagation, and management for 17 agroclimatic zones (p. 552). Nairobi: RELMA in ICRAF Project, World Agroforestry Centre, Eastern Africa Region.
14. Berg, C.C. & Hijman, M.E.E. (1989). Flora of Tropical East Africa, Moraceae: 1-95. CRC Press.
15. Bester, S. P., & Burrows, S. (2015). *Vaccinium exul*. <http://pza.sanbi.org/vaccinium-exul>. Accessed on 10 June 2021.
16. Bidgood, S., Verdcourt, B. & Vollesen, K. (2006). Flora of Tropical East Africa. Bignoniaceae & Cobaeaceae. Kew: Royal Botanic Gardens, Kew.
17. Bingham, M. G., Willemen, A., Wursten, B. T., Ballings, P. and Hyde, M. A. (2022). Flora of Zambia: species information: *Craterispermum schweinfurthii*; *Parkia filicoidea*; *Vernonia syringifolia*; *Rytigynia adenodonta*. <https://www.zambiaflora.com/speciesdata/species.php?species_id=156090>. Accessed 17 April 2022.
18. Bizoux, J. P., Daïnou, K., Bourland, N., Hardy, O. J., Heuertz, M., Mahy, G., & Doucet, J. L. (2009). Spatial genetic structure in *Milicia excelsa* (Moraceae) indicates extensive gene dispersal in a low‐density wind‐pollinated tropical tree. Molecular Ecology, 18 (21), 4398-4408.
19. Boon, R. (2010). Pooley's trees of eastern South Africa, a complete guide. Flora & Fauna Publications Trust, Durban.
20. Bosch, C.H. (2002). *Spathodea campanulata* P. Beauv. <https://www.prota4u.org/database/protav8.asp?g=psk&p=Spathodea%20campanulata>. Accessed 21 June 2022.
21. Bosu, P. P. & Krampah, E. (2005). *Antiaris toxicaria* Lesch Louppe, D. (Ed.). Prota 7: Timbers/Bois d'ceuvre. [CD-Rom]. PROTA, Wageningen, Netherlands.
22. Bradford, J. C., Hopkins, H. C., & Barnes, R. W. (2004). Cunoniaceae. In Flowering Plants· Dicotyledons (pp. 91-111). Springer, Berlin, Heidelberg.
23. Brenan J. P. M. (1967). Leguminosae II. In: Milne-Redhead C. E., Polhill RM (Eds.). Flora of Tropical East Africa. London: Crown Agents.
24. Brenan, J. P. M. (1970). Flora Zambesiaca 3 (1): 1-153. Royal Botanic Gardens, Kew.
25. Bridson, D. M. & Verdcourt, B. (2003). Rubiaceae. Flora Zambesiaca 5:3. Kew: Royal Botanic Gardens, Kew.
26. Bridson, D. M. (1978). Studies in *Pavetta* (Rubiaceae subfam. Cinchonoideae) for part 2
27. Bridson, D. M. (1985). The reinstatement of *Psydrax* (Rubiaceae, subfam. Cinchonoideae tribe Vanguerieae) and a revision of the African species. Kew Bulletin 40 (4):687-725.
28. Bridson, D. M. (1992). The Genus *Canthium* (Rubiaceae: Vanguerieae) in Tropical Africa. Kew Bulletin, 47 (3), 353–401. https://doi.org/10.2307/4110569
29. Bridson, D. M. (1998). Rubiaceae. In: G.V. Pope (Ed.). Flora Zambesiaca 5 (Part 2): 211-369. Royal Botanic Gardens, Kew.
30. Bridson, D. M. (2001). Additional notes on *Pavetta* (Rubiaceae: Pavetteae) from tropical eastern and southern Africa. *Kew Bulletin*, 567-600.
31. Brink, M. (2006). *Craibia brownii* Dunn. In: Brink, M. & Belay, G. (Eds.). PROTA (Plant Resources of Tropical Africa), Wageningen, Netherlands. Accessed 17 April 2022.
32. Brink, M. (2007). *Dombeya rotundifolia* (Hochst.) Planch. PROTA (Plant Resources of Tropical Africa), Wageningen, Netherlands. Accessed 17 April 2022.
33. Brink, M. (2008). *Rhodognaphalon schumannianum* A. Robyns. Record from PROTA4U. Louppe, D., Oteng-Amoako, A.A. & Brink, M. (Eds.). PROTA (Plant Resources of Tropical Africa / Ressources végétales de l’Afrique tropicale), Wageningen, Netherlands. <http://www.prota4u.org/search.asp>. Accessed 17 April 2022.
34. Brown, N. E., Hutchinson, J. and Prain, D. (1925). Flora Capensis, Vol 5, Part 2, page 216.
35. Bruce, E. A. & Lewis, J. (1960). Flora of Tropical East Africa, Loganiaceae: 1-47. [Cited as *Buddleja polystachya*.]
36. Bruce, E. A. & Lewis, J. (1960). Loganiaceae. Flora of Tropical East Africa. CRC Press.
37. Brummitt, R. K., & Marner, S. K. (1993). Flora of Tropical East Africa-Proteaceae (1993). CRC Press.
38. Burkill, H. M. (1985). The Useful Plants of West Tropical Africa (2nd ed.), Whitefriars Press, London.
39. Burrows, J. & Burrows, S. (2003). Figs of southern and south-central Africa. Umdaus Press, Hatfield.
40. Cannon, J. F. M. (1978). Flora Zambesiaca, Vol 4, page 62. Kew: Royal Botanic Gardens, Kew.
41. Carr, J. D. (1988). Combretaceae in southern Africa. Tree Society of Southern Africa, Johannesburg.
42. Chapman, H. M. (2008). The Nigerian Montane Forest Project. Tropinet 19(1), 7-9.
43. Cheek, M. & Dorr, L. (2007). Flora of Tropical East Africa, Sterculiaceae: 1-134. Royal Botanic Gardens, Kew.
44. Child, A. (1998). Studies in *Solanum* and related genera (6). New infrageneric taxa for the genus Solanum L. (Solanaceae). Feddes Repertorium 109, 5-6: 407-427.
45. Chmel, K., Ewome, F. L., Gómez, G. U., Klomberg, Y., Mertens, J. E., Tropek, R., &
46. Cordeiro, N. J., & Howe, H. F. (2001). Low Recruitment of Trees Dispersed by Animals in African Forest Fragments. Conservation Biology, 15 (6), 1733–1741.
47. Cufodontis, R. C. (1966). Pittosporaceae. In: E. Milne-Redhead & R. M. Polhill (Eds.) Flora of Tropical East Africa, London, Crown Agent.
48. Curtis, S. & Viljoen, C. (2010). PlantzAfrica. http://pza.sanbi.org. South African National Biodiversity Institute, Pretoria, South Africa. Accessed on 17 April 2020.
49. Da Silva Vieirasup, M. R., Limasup, G. P. P., de Medeiros, D. C., & de Oliveira, E. C. A. (2012). Genus: Strelitzia. Journal of Horticulture and Forestry, 4 (11), 178-180.
50. Da Silva, G., Serrano, R. & Silva, O. (2011). *Maytenus heterophylla* and *Maytenus senegalensis*, two traditional herbal medicines. Journal of Natural Science, Biology and Medicine 2 (1): 59–65.
51. Davies, F. G., & Verdcourt, B. (1979). Flora of Tropical East Africa - Sapindaceae (1979). CRC Press.
52. Davies, F. G., & Verdcourt, B. (1998). Flora of Tropical East Africa - Sapindaceae (1998). CRC Press.
53. Davies, F. G., & Verdcourt, B. (1998). Flora of Tropical East Africa - Sapindaceae. CRC Press.
54. Davis, A. P., Govaerts, R., Bridson, D. M., & Stoffelen, P. (2006). An annotated taxonomic conspectus of the genus *Coffea* (Rubiaceae). Botanical Journal of the Linnean Society, 152 (4), 465-512.
55. De Winter, B. (1966). Sixty-six Transvaal Trees, Pretoria, Botanical Research Institute, Department of Agricultural Technical Services.
56. Dominy, N. J., & Duncan, B. W. (2005). Seed-spitting primates and the conservation and dispersion of large-seeded trees. International Journal of Primatology, 26 (3), 631-649.
57. Dressler S, Schmidt M, Zizka G. (2014). African Plants - A Photo Guide. www.africanplants.senckenberg.de. Forschungsinstitut Senckenberg, Frankfurt/Main, Germany. Accessed on 21 June 2021.
58. Dyer, R. A. (1943). The genus *Lachnostylis* (Turcz.). South African Journal of Science, 40 (11), 123-126.
59. Exell, A.W. (1966). Sapindaceae. Flora Zambesiaca 2 (2). Pages 502 - 503
60. Exell, A.W. (1978). Combretaceae. Flora Zambesiaca 4. Page 179.
61. Fernandes, R. & Fernandes, A. (1966). Anacardiaceae. Flora Zambesiaca 2:2.
62. Fernandes, R. (2005). Lamiaceae (subfamilies: Viticoideae and Ajugoideae). Flora Zambesiaca 8 (7) Pages 104 - 108.
63. Flora Somalia, Vol 3, (2006) Author: by D. Bridson & M. Thulin (Pavetta)
64. Foden, W. & Potter, L. (2005). *Seemannaralia gerrardii* (Seem.) Harms. National Assessment: Red List of South African Plants version 2020.1. Accessed on 17 April 2022.
65. Friis, I. (1989). Urticaceae. Flora of Tropical East Africa. University of Copenhagen, Denmark.
66. Friis, I. (1993). Rhizophoraceae. In: Thulin, M. (Editor). Flora of Somalia. Volume 1. Pteridophyta; Gymnospermae; Angiospermae (Annonaceae-Fabaceae). Royal Botanic Gardens, Kew, Richmond, United Kingdom. pp. 254–258.
67. GBIF.org (01 October 2021) GBIF Occurrence Download https://doi.org/10.15468/dl.gdwn8w
68. GBIF.org (10 October 2021) GBIF Occurrence Download https://doi.org/10.15468/dl.ae7kta
69. Geldenhuys, C. J. (1993). Floristic composition of the southern Cape forests with an annotated check-list. South African Journal of Botany, 59 (1), 26-44.
70. Geldenhuys, C. J. (1996). Forest management systems to sustain resource use and biodiversity: examples from the southern Cape, South Africa. In The biodiversity of African plants (pp. 317-322). Springer, Dordrecht.
71. Geldenhuys, C. J. (1997). Composition and biogeography of forest patches on the inland mountains of the southern Cape. Bothalia, 27 (1), 57-74.
72. Gillett, J. B. (1991). Burseraceae. Flora of Tropical East Africa.
73. Gillett, J. B., Polhill, R. M., & Verdcourt, B. (1971). Leguminosae. Flora of Tropical East Africa.
74. Gonçalves A. E. (2005). Solanaceae. Flora Zambesiaca. Vol. 8, Part 4.
75. Govaerts, R. (2003). World Checklist of Selected Plant Families Database in ACCESS: 1-216203. The Board of Trustees of the Royal Botanic Gardens, Kew
76. Govaerts, R., Frodin, D.G. & Radcliffe-Smith, A. (2000). World Checklist and Bibliography of Euphorbiaceae (and Pandaceae) 1-4: 1-1622. The Board of Trustees of the Royal Botanic Gardens, Kew
77. Govaerts, R., Nic Lughadha, E., Black, N., Turner, R. & Paton, A. (2021). The World Checklist of Vascular Plants, a continuously updated resource for exploring global plant diversity. https://doi.org/10.1038/s41597-021-00997-6 Scientific Data 8: 215.
78. Graham, R. A. (1960) Rosaceae. Flora of Tropical East Africa.
79. Grant, R. & Thomas, V. (2001). Sappi tree spotting: Lowveld including Kruger National Park. Jacana, Johannesburg.
80. Gunn, M. & Codd, L.E. (1981). Botanical exploration of southern Africa. Balkema, Cape Town.
81. Hardy, O. J., Delaide, B., Hainaut, H., Gillet, J. F., Gillet, P., Kaymak, E., & Doucet, J. L. (2019). Seed and pollen dispersal distances in two African legume timber trees and their reproductive potential under selective logging. Molecular ecology, 28 (12), 3119-3134.
82. Harley, R. M., Atkins, S., Budantsev, A. L., Cantino, P. D., Conn, B. J., Grayer, R., Harley, M. M., de Kok, R. P. J., Krestovskaja, T., Morales, R., Paton, A. J., Ryding, O. & Upson, T. (2004). Labiatae. In: K. Kubitzki & J. W. Kadereit (Eds.), The Families and Genera of Vascular Plants vol. VII:167 – 275. Springer-Verlag, Berlin.
83. Harrington, M. (2008). Phylogeny and evolutionary history of Sapindaceae and Dodonaea (Doctoral dissertation, James Cook University).
84. Hassan, A. S. & Cheek, M. (1999). Meliaceae. In: M. Thulin (Ed.), Flora of Somalia 2, pp. 228-238. Trustees of the Royal Botanic Gardens, Kew, Richmond, Surrey, UK.
85. Hemsley, J. H. (1958). Flora of Tropical East Africa.
86. Herman, P. P. J. (2002). Revision of the *Tarchonanthus camphoratus* complex (Asteraceae-Tarchonantheae) in southern Africa. Bothalia 32: 21-28.
87. https://indiabiodiversity.org/species/show/12638
88. https://worldofsucculents.com/aloe-ballyi-rat-aloe/
89. Hyde, M. A., Wursten, B. T., Ballings, P. & Coates Palgrave, M. (2022). Flora of Zimbabwe: Home page. https://www.zimbabweflora.co.zw/index.php, retrieved 18 April 2022
90. identification, propagation and management for agricultural and pastoral communities. Regional
91. Janeček, Š. (2021). Bird pollination syndrome is the plant's adaptation to ornithophily, but nectarivorous birds are not so selective. Oikos, 130 (8), 1411-1424.
92. Joffe, P. (1993). The gardener's guide to South African plants. Tafelberg, Cape Town.
93. Jordaan, M. & van Wyk, A. E. (2006). Sectional classification of Gymnosporia (Celastraceae), with notes on the nomenclatural and taxonomic history of the genus. Taxon 55 (2): 515–525.
94. Jordaan, M. (2012). Iteaceae. Bothalia, 42 (2), 196-200.
95. Kaiser-Bunbury, C. N., Memmott, J., & Müller, C. B. (2009). Community structure of pollination webs of Mauritian heathland habitats. Perspectives in Plant Ecology, Evolution and Systematics, 11 (4), 241-254.
96. Katende, A. B., Birnie, A., Tegnas, B. (1995). Useful Trees and Shrubs for Uganda,
97. Keay, R. W. J. (1959) An outline of Nigerian vegetation, ed. 3. La Federal Government Printer.
98. Knopf, P., Schulz, C., Little, D. P., Stützel, T., & Stevenson, D. W. (2012). Relationships within Podocarpaceae based on DNA sequence, anatomical, morphological, and biogeographical data. Cladistics, 28 (3), 271-299.
99. Knox, E. B., & Palmer, J. D. (1995). Chloroplast DNA variation and the recent radiation of the giant senecios (Asteraceae) on the tall mountains of eastern Africa. Proceedings of the National Academy of Sciences, 92 (22), 10349-10353.
100. Kochummen, K. M. (1995). *Gomphia* (Schreb.). In Soepadmo, E.; Wong, K. M. (Eds.). Tree Flora of Sabah and Sarawak. Vol. 1. Forest Research Institute Malaysia. pp. 265 - 267.
101. Kokwaro, J. O. (1982). Flora of Tropical East Africa, Rutaceae: 1-52.
102. Kokwaro, J. O. (1986). Flora of Tropical East Africa, Anacardiaceae: 1-59.
103. Kudoh, H., Shimamura, R., Takayama, K., & Whigham, D. F. (2006). Consequences of hydrochory in *Hibiscus*. Plant Species Biology, 21 (3), 127-133.
104. Kupicha, F. K. (1978). Flora Zambesiaca 4. Caricaceae.
105. Kupicha, F. K. (1983). Flora Zambesiaca 7:1. Sapotaceae.
106. Le Roux, M. M., Van Wyk, B. E., Boatwright, J. S., & Tilney, P. M. (2011). The systematic significance of morphological and anatomical variation in fruits of *Crotalaria* and related genera of tribe Crotalarieae (Fabaceae). Botanical Journal of the Linnean Society, 165 (1), 84-106.
107. Lemmens, R. H. M. J. (2007). *Pouteria adolfi-friedericii* (Engl.) A. Meeuse. Record from PROTA4U. Louppe, D., Oteng-Amoako, A.A. & Brink, M. (Eds). PROTA (Plant Resources of Tropical Africa / Ressources végétales de l’Afrique tropicale), Wageningen, Netherlands. <http://www.prota4u.org/search.asp>. Accessed 18 April 2022.
108. Lewis, G. P., Schrire, B., Mackinder, M. L. (2005). Legumes of The World. Royal Botanic Gardens, Kew, London, United Kingdom.
109. Lewis, J. (1960). Flora Zambesiaca 1:1. Cupressaceae.
110. Lovett, J.C. (1993). Eastern Arc moist forest flora. In J.C. Lovett & S.K. Wasser (Eds.) Biogeography and Ecology of the Rain Forests of Eastern Africa. University Press, Cambridge, 33–55.
111. Mackinder, B., Pasquet, R., Polhill, R. & Verdcourt, B. (2001). Flora Zambesiaca 3 (5): 1-261. Royal Botanic Gardens, Kew.
112. Marner, S. K. (1989). New and noteworthy species of *Faurea* (Proteaceae) from the evergreen forests of Malawi and elsewhere. Bulletin du Jardin botanique national de Belgique / Bulletin van de Nationale Plantentuin van Belgie, 427-431.
113. Maroyi, A. (2012). *Markhamia lutea* (Benth.) K. Schum. Record from PROTA4U. Lemmens, R. H. M. J., Louppe, D. & Oteng-Amoako, A. A. (Eds). PROTA (Plant Resources of Tropical Africa / Ressources végétales de l’Afrique tropicale), Wageningen, Netherlands. <http://www.prota4u.org/search.asp>. Accessed 18 April 2022.
114. Mbambezeli, G. & Notten, A. (2014). *Schotia brachypetala* Sond. (Fabaceae). PlantZAfrica. Online. <http://pza.sanbi.org/schotia-brachypetala>. Accessed 18 April 2022.
115. Mendes, E. J. (1963). Flora Zambesiaca, Vol 2, Part 1, page 340.
116. Mendes, E. J. (1978). Rosaceae. Flora Zambesiaca, 4, pages 21 – 22.
117. Mng'omba, S. A., Du Toit, E. S., Akinnifesi, F. K. and Venter, H. M. (2007). Repeated exposure of jacket plum (*Pappea capensis*) micro-cuttings to indole-3-butyric acid (IBA) improved in vitro rooting capacity. South Afr. J. Bot., 73: 230-235.
118. Moffett, R. (2007). Name changes in the Old World *Rhus* and recognition of *Searsia* (Anacardiaceae). Bothalia, 37 (2), 165-175. https://doi.org/10.4102/abc.v37i2.311.
119. Mollel, N. P. (2013). *Maerua triphylla* A .Rich. In: Schmelzer, G.H. & Gurib-Fakim, A. (Eds). Prota 11(2): Medicinal plants/Plantes médicinales 2. PROTA, Wageningen, Netherlands. Accessed 18 April 2022.
120. Mosango, D. M. (2007). *Mallotus oppositifolius* (Geiseler) Müll.Arg. Record from PROTA4U. Schmelzer, G.H. & Gurib-Fakim, A. (Eds). PROTA (Plant Resources of Tropical Africa / Ressources végétales de l’Afrique tropicale), Wageningen, Netherlands. <http://www.prota4u.org/search.asp>. Accessed 18 April 2022.
121. Muchane, M. N. (2019). Population Status, Distribution Patterns and Conservation Needs of Endangered *Croton alienus* Pax in Kenya. International Journal of Natural Resource Ecology and Management. 120 – 128.
122. Mugo, S. M., & Njenga, P. K. (2020). Chemical composition, antioxidant potential and antimicrobial activities of *Ixora scheffleri* subspecies *keniensis* essential oil. Journal of Medicinal Plants for Economic Development, 4 (1), 1-7.
123. Munzinger, J. & Pauly, A. (2003). Mechanism of self pollination in *Hybanthus enneaspermus* (L.) F. Muell. and notes on the floral biology of some *Rinorea* species (Violaceae) in Ivory Coast. Acta Botanica Gallica 150(2): 155-166.
124. Mwachala, G. & Mbugua P. K. (2007). Dracaenaceae. In: Beentje, H. J., Ghazanfar, S. A. (Eds), Flora of tropical East Africa. Royal Botanic Gardens, Kew, London.
125. Mwavu, E. N., & Witkowski, E. T. (2009). Population structure and regeneration of multiple-use tree species in a semi-deciduous African tropical rainforest: Implications for primate conservation. Forest Ecology and Management, 258 (5), 840-849.
126. Nesom, G. L. (1990). Further definition of *Conyza* (Asteraceae: Astereae). Phytologia, 68, 229-233.
127. Nyemb, N. (2011). *Mitragyna stipulosa* (DC.) Kuntze. Record from PROTA4U. Lemmens, R.H.M.J., Louppe, D. & Oteng-Amoako, A.A. (Editors). PROTA (Plant Resources of Tropical Africa / Ressources végétales de l’Afrique tropicale), Wageningen, Netherlands. <http://www.prota4u.org/search.asp>. Accessed 18 April 2022.
128. of Flora of Tropical East Africa. Kew Bull. 32: 60.
129. Omino, E. A. (2002). Apocynaceae. Flora of Tropical East Africa.
130. Onyekwelu, J. C., & Stimm, B. (2004). *Treculia africana*. Enzyklopädie der Holzgewächse: Handbuch und Atlas der Dendrologie, 1-12.
131. Orwa, C., Mutua, A., Kindt, R., Jamnadass, R., Anthony, S. (2009). Agroforestree Database: a tree reference and selection guide version 4.0. World Agroforestry Centre, Kenya.
132. Oskolski, A. A., Sokoloff, D. D., & Van Wyk, B. E. (2010). False paracarpy in *Seemannaralia* (Araliaceae): from bilocular ovary to unilocular fruit. Annals of botany, 106 (1), 29-36.
133. Osunkoya, O. O. (1999). Population structure and breeding biology in relation to conservation in the dioecious *Gardenia actinocarpa* (Rubiaceae) - a rare shrub of North Queensland rainforest. Biological Conservation. 88, 347–359.
134. Oyen, L. P. A. & Lemmens, R. H. M. J. (Eds.). PROTA (Plant Resources of Tropical Africa / Resources végétales de l’Afrique tropicale), Wageningen, Netherlands. <http://www.prota4u.org/search.asp>. Accessed 17 April 2022.
135. Oyen, L. P. A. (2011). *Ficus natalensis* Hochst. Record from PROTA4U. Brink, M. & Achigan-Dako, E.G. (Editors). PROTA (Plant Resources of Tropical Africa / Ressources végétales de l’Afrique tropicale), Wageningen, Netherlands. <http://www.prota4u.org/search.asp>. Accessed 18 April 2022.
136. Palgrave, K. C. (1977). Trees of South Africa. C. Struik Publishers. Cape Town, Johannesburg.
137. Palgrave, K. C. (2013). Palgrave's trees of southern Africa. Penguin Random House South Africa.
138. Palgrave, M. C. (2002). Trees of Southern Africa. Cape Town: Struik Publishers.
139. Palmer, E., Pitman, N., & Codd, L. E. W. (1972). Trees of southern Africa covering all known indigenous species in the Republic of South Africa, South-West Africa, Botswana, Lesotho and Swaziland. Vol. 1. A. A. Balkema, Cape Town.
140. Pell, S. K. (2004). Molecular systematics of the cashew family (Anacardiaceae). Louisiana State University and Agricultural & Mechanical College.
141. Peterson, B. (1978). University of Göteborg. Thymelaeaceae. Flora of Tropical East Africa.
142. Pfeil, B. E., & Crisp, M. D. (2005). What to do with *Hibiscus*? A proposed nomenclatural resolution for a large and well known genus of Malvaceae and comments on paraphyly. Australian Systematic Botany, 18 (1), 49-60.
143. Plants for a Future (2010). https://pfaf.org/user/Plant.aspx?LatinName=Vitex+keniensis
144. Polhill, R. M., & Verdcourt, B. (2000). Flora of Tropical East Africa. Myricaceae. CRC Press.
145. Pooley, E. (2003). The complete field guide to trees of Natal, Zululand and Transkei. Natal Flora Publication trust. 188 - 189.
146. Potgieter, M. J., & Van Wyk, A. E. (1994). Fruit structure of the genus *Cassinopsis* Sond. (Icacinaceae) in Africa. South African Journal of Botany, 60 (2), 117-122.
147. Prance, G. T. (1975). The correct name for Castanha de cutia (*Couepia edulis* (Prance) Prance-Chrysobalanaceae). Acta amazônica, 5, 143-145.
148. Radcliffe-Smith, A. (1996). Euphorbiaceae. Flora Zambesiaca 9:4.
149. Rangaiah, K., Purnachandra Rao, S., & Solomon Raju, A. J. (2004). Bird-pollination and fruiting phenology in *Spathodea campanulata* Beauv. (Bignoniaceae). Beitrage zur Biologie der Pflanzen, 73 (3), 395.
150. Rasoloarijao, T. M., Ramavovololona, P., Ramamonjisoa, R., Clemencet, J., Lebreton, G., & Delatte, H. (2019). Pollen morphology of melliferous plants for *Apis mellifera unicolor* in the tropical rainforest of Ranomafana National Park, Madagascar. Palynology, 43 (2), 292-320.
151. Robbrecht, E. (1987). The African genus *Tricalysia* A. Rich. (Rubiaceae). 4. A revision of the species of section *Tricalysia* and section Rosea. Bulletin du Jardin botanique national de Belgique / Bulletin van de Nationale Plantentuin van Belgie, 39-208.
152. Robbrecht, E., & De Block, P. (2002). 447. *Didymosalpinx norae*: Rubiaceae. Curtis’s Botanical Magazine, 19 (3), 165–172. http://www.jstor.org/stable/45065541
153. Robson, N. K. B. (1960). Annonaceae. Flora Zambesiaca 1:1.
154. Robson, N. K. B. (1961). Guttiferae. Flora Zambesiaca 1:2.
155. Robson, N. K. B. (1966). Celastraceae. Flora Zambesiaca 2:2.
156. Rourke, J. P. (1998). A review of the systematics and phylogeny of the African Proteaceae. Australian Systematic Botany, 11 (4), 267-285.
157. Roux, J. P. (2003). Flora of South Africa. SANBI, Cape Town.
158. Rudd, V. E. (1955). The American species of *Aeschynomene*. Leguminosae.
159. Saunders, R. M. (2012). The diversity and evolution of pollination systems in Annonaceae. Botanical Journal of the Linnean Society, 169 (1), 222-244.
160. Schmidt, E., Lötter, M. & McCleland, W. (2002). Trees and shrubs of Mpumalanga and Kruger National Park. Jacana, Johannesburg.
161. Sebola, R. J., & Balkwill, K. (2009). Numerical phenetic analysis of *Olinia rochetiana* sensu lato (Oliniaceae). Kew Bulletin, 64 (1), 95-121.
162. Sinou, C., Forest, F., Lewis, G. P., & Bruneau, A. (2009). The genus *Bauhinia* sl (Leguminosae): a phylogeny based on the plastid trn L–trn F region. Botany, 87 (10), 947-960.
163. Sleumer, H. (1975). Flora of Tropical East Africa, Flacourtiaceae: 1-68.
164. Sleumer, H. (1975). Flora of Tropical East Africa, Flacourtiaceae: 1-68.
165. Smedmark, J. E., Razafimandimbison, S. G., Wikström, N., & Bremer, B. (2014). Inferring geographic range evolution of a pantropical tribe in the coffee family (Lasiantheae, Rubiaceae) in the face of topological uncertainty. Molecular phylogenetics and evolution, 70, 182-194.
166. Smith, A. P., & Young, T. P. (1982). The cost of reproduction in *Senecio keniodendron*, a giant rosette species of Mt. Kenya. Oecologia, 55 (2), 243-247.
167. Smith, A. R. (1987). Macaranga. Flora of Tropical East Africa. Royal Botanic Gardens, Kew and East African Herbarium, 239-251.
168. soil conservation unit (RSCU), Nairobi.
169. Ssali, F., Moe, S. R., & Sheil, D. (2018). Tree seed rain and seed removal, but not the seed bank, impede forest recovery in bracken (*Pteridium aquilinum* (L.) Kuhn) ‐ dominated clearings in the African highlands. Ecology and Evolution, 8 (8), 4224-4236.
170. Styles, B. T. & F. White, F. (1991). Department of Plant Sciences, University of Oxford. Meliaceae. Flora of Tropical East Africa.
171. Styles, B.T. & White, F. (1991). Flora of Tropical East Africa, Meliaceae: 1-67. Royal Botanic Gardens, Kew.
172. Sunnichan, V. G., Mohan Ram, H. Y., & Shivanna, K. R. (2004). Floral sexuality and breeding system in gum karaya tree, *Sterculia urens*. Plant Systematics and Evolution, 244 (3), 201-218.
173. Swelankomo, N., Manning, J. C., & Magee, A. R. (2016). The genus *Gymnanthemum* Cass. (Asteraceae: Vernonieae) in Southern Africa. South African Journal of Botany, 102, 81-101.
174. Tchouto, M. G. P., De Wilde, J. J. F. E., De Boer, W. F., Van der Maesen, L. J. G., & Cleef, A. M. (2009). Bio-indicator species and Central African rain forest refuges in the Campo-Ma'an area, Cameroon. Systematics and Biodiversity, 7 (1), 21-31.
175. Tennant, J. (1960). Notes on African Araliaceae. II. Kew Bulletin, 13, 400–401.
176. Thiele, K. R., Funk, V. A., Iwatsuki, K., Morat, P., Peng, C. I., Raven, P. H., ... & David, J. C. (2011). The controversy over the retypification of *Acacia* Mill. with an Australian type: a pragmatic view. Taxon, 60 (1), 194-198.
177. Thijs, K. W., Roelen, I., & Musila, W. M. (2014). Field guide to the woody plants of Taita hills, Kenya. Journal of East African Natural History, 102 (1-2), 1-272.
178. Thomas, V and Grant, R. (1998). Sappi Tree Spotting, Highveld and the Drakensberg. Jacana Education. Johannesburg.
179. Thompson, D. I., & Edwards, T. J. (2001). Breeding biology, resource partitioning and reproductive effort of a dioecious shrub, *Clutia pulchella* L. (Euphorbiaceae). Plant Systematics and Evolution, 226 (1), 13-22.
180. Thulin, M. (1983). Lobeliaceae. Flora Zambesiaca 7 (1). Pages 120 – 122.
181. Thulin, M. (Ed.) (1993). Flora of Somalia 1: 1-493. The Royal Botanic Gardens, Kew
182. Thulin, M. (Ed.) (2006). Flora of Somalia 3: 1-626. The Royal Botanic Gardens, Kew
183. Thulin, M. (Ed.) (2008). Flora of Somalia 2: 1-303. The Royal Botanic Gardens, Kew.
184. Tosh, J., Dessein, S., Buerki, S., Groeninckx, I., Mouly, A., Bremer, B., & De Block, P. (2013). Evolutionary history of the Afro-Madagascan *Ixora* species (Rubiaceae): species diversification and distribution of key morphological traits inferred from dated molecular phylogenetic trees. Annals of botany, 112 (9), 1723-1742.
185. Tropical Plants Database, Ken Fern. 2022-04-19. <tropical.theferns.info/viewtropical.php?id=Vitex+keniensis>
186. Turrill, W. B., & Milne-Redhead, E. (1952). Flora of tropical East Africa. Flora of tropical East Africa. Royal Botanic Gardens, Kew.
187. Uhl, N. W., & Moore Jr, H. E. (1977). Correlations of inflorescence, flower structure, and floral anatomy with pollination in some palms. Biotropica, 170-190.
188. Van Velzen, R., Wahlert, G. A., Sosef, M. S., Onstein, R. E., & Bakker, F. T. (2015). Phylogenetics of African *Rinorea* (Violaceae): Elucidating infrageneric relationships using plastid and nuclear DNA sequences. Systematic Botany, 40 (1), 174-184.
189. Van Wyk, A. E. & Van Wyk, P. (1997). Field guide to trees of southern Africa: 418 - 419. Struik, Cape Town.
190. Van Wyk, P. (1984). Field Guide to the trees of the Kruger Nationl Park, Cape Town, Struik Publishers.
191. Venter, E. (2011). Trees of the Garden Route – Mossel Bay to Storms River. Briza Publishing, Pretoria.
192. Verdcourt, B. (1958). Canellaceae. Flora of Tropical East Africa. Royal Botanic Gardens, Kew.
193. Verdcourt, B. (1971). Annonaceae. Flora of Tropical East Africa. Royal Botanic Gardens, Kew.
194. Verdcourt, B. (1975). Flora of Tropical East Africa. Royal Botanic Gardens, Kew.
195. Verdcourt, B. (1976). Rubiaceae. Flora of Tropical East Africa. Royal Botanic Gardens, Kew.
196. Verdcourt, B. (1978). Oliniaceae. In: E. Launert (Ed.), Flora Zambesiaca 4: 323 – 327. Flora Zambesiaca Managing Committee, London.
197. Verdcourt, B. (1984). Erythroxylaceae. Flora of Tropical East Africa. Royal Botanic Gardens, Kew.
198. Verdcourt, B. (1989). Rubiaceae. Flora Zambesiaca 5:1.
199. Verdcourt, B. (1991). Flora of Tropical East Africa, Boraginaceae: 1-124.
200. Verdcourt, B. (2000). Flora Zambesiaca 3 (6): 1-175. Royal Botanic Gardens, Kew.
201. Verdcourt, B. (2001). Myrtaceae. In: Beentje, H.J. (Ed.). Flora of Tropical East Africa. A. A. Balkema, Rotterdam, Netherlands. 89 pp.
202. Verdoon, I. C., & Herman, P. P. J. (1986). Revision of the genus *Dombeya* (Sterculiaceae) in southern Africa. Bothalia, 16 (1), 1-9.
203. Vollesen, K. B. (2008). Acanthacae. Pages 1–286 in Beentje, H. J., & Ghazanfar, S. A. (Eds.) Flora of tropical East Africa. Pt 1. Royal Botanic Gardens, Kew.
204. Von Breitenbach, F. V. (1974). Southern Cape Forests and Trees. Government Printer, Pretoria.
205. Von Breitenbach, F. V. (1985). Southern Cape Tree Guide. Department of Environmental Affairs, Forestry Branch, Pretoria.
206. Vorontsova, M. S., & Mbago, F. M. (2010). New Solanum species from Tanzanian coastal forests may already be extinct. Journal of East African Natural History, 99 (2), 227-234.
207. Webster, G. L. (1984). A revision of Flueggea (Euphorbiaceae). Allertonia, 3 (4), 259-312.
208. White, F. (1966). Melianthaceae. Flora Zambesiaca 2 (2). Pages 546 – 547. Royal Botanic Gardens, Kew.
209. White, F. (1978). Chrysobalanaceae. Flora Zambesiaca 4. Pages 43 – 44. Royal Botanic Gardens, Kew.
210. White, F., & Styles, B. T. (1963). Flora Zambesiaca. Royal Botanic Gardens, Kew.
211. White, F., & Verdcourt, B. (1996). Flora of Tropical East Africa - Ebenaceae. CRC Press.
212. White, F., Dowsett-Lemaire, F., & Chapman, J. D. (2001). Evergreen forest flora of Malawi. Royal Botanic Gardens.
213. Whitehouse, C. (2001). *Grewia* In Whitehouse, C., Cheek, M., Andrews, S. and Verdcourt, B. Tiliaceae. Flora of Tropical East Africa.
214. Wickens, G. E. (1975). Melastomataceae. Flora of Tropical East Africa. Royal Botanic Gardens, Kew.
215. Wild, H. (1960). Capparidaceae. Flora Zambesiaca 1:1.
216. Wild, H. (1960). Flacourtiaceae. Flora Zambesiaca 1:1. Royal Botanic Gardens, Kew.
217. Wilmot-Dear, C.M. (1991). Ulmaceae. Flora Zambesiaca 9 (6). Pages 8 - 10. Royal Botanic Gardens, Kew.
218. Wright, C. H. (1905). Flora of Tropical Africa, Vol IV, Part 2, page 207. Royal Botanic Gardens, Kew.
219. Zhang, X., Zhang, Z., & Stützel, T. (2014). Ontogeny of the ovule and seed wing in *Catha edulis* (Vahl) Endl.(Celastraceae). Flora-Morphology, Distribution, Functional Ecology of Plants, 209(3-4), 179-184.
220. Zietsman, P. C. (1991). Reproductive biology of *Grewia occidentalis* L. (Tiliaceae). South African Journal of Botany, 57 (6), 348-351.
